# Effects of erythropoietin administration on allogeneic blood transfusion requirements in adults undergoing surgery: a systematic review and meta-analysis

**DOI:** 10.3389/fmed.2025.1712121

**Published:** 2026-01-14

**Authors:** Jia Chen, Bo Liu, Huixiu Cao, Tao Tian, Jie He

**Affiliations:** 1 Department of Laboratory Medicine, Chongqing Jiangjin District Second People's Hospital, Chongqing, China; 2 Department of Urology, Chongqing Jiangjin District Second People's Hospital, Chongqing, China

**Keywords:** erythropoietin, surgical procedures, blood transfusion, adult, randomized controlled trial

## Abstract

**Background:**

Erythropoietin is commonly integrated into blood management strategies for surgical patients, yet its administration may lead to adverse outcomes. This systematic review and meta-analysis assesses the impact of erythropoietin administration on perioperative transfusion requirements and adverse events.

**Methods:**

A comprehensive search across four databases was conducted from inception to 30 July 2025 to identify randomized controlled trials involving adult surgical patients. The methodological quality of the included studies was evaluated using the Cochrane Risk of Bias Assessment Tool. The outcome measures included transfusion rates, volume of allogeneic red blood cell transfusions per patient unit, mortality rates, postoperative infection rates, transfusion-related complications, venous thromboembolism rates, and length of hospital stay. Effect sizes were reported using relative risk (RR) and standardized mean difference (SMD). We used GRADE to assess the quality of evidence for each outcome.

**Results:**

Preoperative erythropoietin administration reduces transfusion rates (RR = 0.63, 95% CI = 0.53–0.75, *p* = 0.000) and mean transfusion volumes (SMD = −0.33, 95% CI = −0.42 to −0.24, *p* = 0.000) in surgical patients. No significant impact was observed on mortality, postoperative infection incidence, postoperative complications, adverse reactions, venous thromboembolic events, and length of hospital stay. Subgroup analyses showed significant reductions in transfusion rates for cardiac surgery, orthopedic surgery, gastrointestinal surgery, gynecological abdominal surgery, and other types of surgery.

**Conclusion:**

The available evidence supports the efficacy of preoperative human erythropoietin administration in reducing transfusion requirements and the average number of transfusion units in adult surgical patients. No significant differences were observed in postoperative mortality, complication rate, adverse event rate, venous thromboembolic events (including deep vein thrombosis and pulmonary embolism), and hospital stay after preoperative administration of erythropoietin.

## 1 Introduction

In 2012, global surgical procedures totaled an estimated 312.9 million, with an average global surgery rate of 4,469 operations per 100,000 individuals annually (1). Bleeding events are common during surgeries, necessitating transfusion therapy to enhance blood oxygen-carrying capacity and tissue oxygenation through the intravenous infusion of blood components. Research indicates that 8%−36% of patients undergoing spinal surgery require perioperative blood transfusions, typically administered within a window spanning 7 days before to 30 days after the procedure (2).

In regions with robust medical infrastructure, major surgery (61.2%) is the primary indication for massive transfusion, followed by trauma (15.4%) (3). While blood transfusions play a crucial role in stabilizing patients' physiological status, their excessive use can lead to adverse effects. Perioperative bleeding, among various patient and surgical factors, contributes significantly to complications and mortality rates (4). Erythropoietin, a hormone synthesized by the kidneys, stimulates the production of red blood cells in the bone marrow. Erythropoiesis-stimulating agents (ESAs) have been used in patients undergoing surgery to reduce or eliminate the need for allogeneic blood transfusions (5). Erythropoietic interventions are often integrated into blood management protocols for surgical patients. Nevertheless, the utilization of erythropoietin may have negative consequences. Although several studies have explored the correlation between erythropoietin administration and outcomes in orthopedic, cardiac, and other surgery types, the impact of erythropoietic interventions on blood transfusion in surgical patients is still controversial. A meta-analysis conducted in 2019 synthesized the findings of randomized controlled trials (RCTs) investigating the preoperative administration of erythropoietin stimulants in surgical patients (6). This analysis, encompassing studies from 1993 to 2018, concluded that preoperative erythropoietin use correlated with a decreased incidence of allogeneic transfusions (6). Our research also corroborates this outcome.

Given the advancements in surgical practices and the evolving body of evidence since the publication of the 2019 meta-analysis, further investigations are warranted to evaluate the contemporary relevance of erythropoietin use in clinical settings. By systematically reviewing and analyzing recent trials, we expanded upon the aforementioned meta-analysis to include parameters such as average transfusion volume, mortality rates, and infection rates, among others, with the aim of providing more precise and conclusive insights. The objective of the present study is to expand the overall value of ESAs in perioperative care with the goal of minimizing or obviating the need for transfusion and improving patient outcomes.

## 2 Materials and methods

### 2.1 Protocol

This systematic review was conducted in accordance with the Preferred Reporting Items for Systematic Reviews and Meta-Analyses for Meta-Analyses (PRISMA; Supplementary Table S3) and has been registered with PROSPERO, a prospective systematic review registration platform operated by the National Institute for Health and Care Research in the UK, under the registration number CRD420251126117.

### 2.2 Search strategy

The researchers conducted a systematic search of PubMed, Embase, Web of Science, and Cochrane for studies available from inception to 30 July 2025, without restrictions on publication language or status. They also reviewed references of relevant articles to identify additional eligible studies. Detailed search strategies are available in Supplementary File S1.

### 2.3 Inclusion and exclusion criteria

#### 2.3.1 Types of participants

We included trials with adult subjects (age ≥18 years prior to enrollment)—regardless of gender and type of surgery, with no minimum sample size or specific dosing regimen required, allowing for the inclusion of various doses—who received emergency or elective surgery.

#### 2.3.2 Types of interventions

We incorporated all preoperative trials involving the administration of recombinant human erythropoietin, with or without iron supplementation, initiated at any point between the decision to proceed with surgery and the day of the operation. The administration of recombinant human erythropoietin encompassed varying doses, routes (subcutaneous or intravenous), and durations. The trials under consideration compared the efficacy of EPO treatment against that of control interventions such as placebo, no treatment, or standard treatment (including iron supplementation).

#### 2.3.3 Outcomes

##### primary-outcome

The primary outcome was the perioperative allogeneic red blood cell transfusion rate, excluding autologous transfusions and transfusions of other blood products such as platelets and plasma.

##### secondary-outcomes

The secondary outcomes were as follows:

a) The number of allogeneic red blood cell transfusions (units/patient);
b) Mortality;
c) Postoperative infection rate;
d) Postoperative complications and adverse effects;
e) Venous thromboembolic events (VTE) rate [deep vein thrombosis (DVT)/pulmonary embolism (PE)];
f) Hospital length of stay (days).

#### 2.3.4 Study design

Randomized controlled trials (RCTs) published in full text in any language were included.

#### 2.3.5 Exclusion criteria

Studies reporting the following were excluded: (1) animal experiments, meta-analyses, conference abstracts, systems reviews, reviews, letters, and case reports; and (2) non-randomized controlled trials or studies where full text was not available.

#### 2.3.6 Study selection and data extraction

Two reviewers independently screened all citations and abstracts to ascertain relevance. Full trial reports were retrieved where the studies were considered potentially relevant. If not, full texts were retrieved to determine eligibility. Two reviewers independently screened the trial reports and made decisions on which reports to include, and disagreements resolved by consensus. Data from these chosen studies were collected with a standardized form.

Two reviewers collected all data. The following data were collected: first author; publication year; sample size for both the experimental and control groups; percentage of male subjects; age distribution; EPO intervention database information related to doses; administration frequency; and duration; EPO type (rhEPO or darbepoetin-α supplementation); and intervention protocols (time, frequency, and periodicity) with respective return intervals between endpoint measurement assessments as well concomitant procedures recorded. When deemed necessary, we contacted the corresponding author of each study via the email address provided in the article to obtain additional detailed information regarding missing or incomplete data. The two researchers conducted it independently, and the differences were resolved through consensus or arbitration by a third researcher.

#### 2.3.7 Quality assessment

In accordance with the guidelines for evidence-based medicine, our two reviewers used the Cochrane Risk of Bias Assessment tool to assess study quality and evaluated the studies according to six criteria: random sequence generation, allocation concealment, blinding (assessment of risk), blinding (detection of bias), incomplete outcome data, selective reporting, and other bias sources. Any differences among researchers are resolved through discussion. Subsequently, according to the criteria of the tool, each study was classified as having a low risk of bias, a high risk of bias, or an uncertain risk.

In addition, we employed the GRADE approach to assess the quality of evidence across predetermined outcomes. Initially, evidence from randomized controlled trials began at a high-quality grade, while evidence from observational studies commenced at a low-quality grade. Subsequently, we systematically lowered the evidence quality based on five criteria: risk of bias, inconsistency, indirectness, imprecision, and publication bias. In the case of observational studies, additional factors such as effect size, dose-response relationships, and potential confounding variables were considered as escalating conditions. The evaluation was conducted independently by two researchers, with any discrepancies resolved through discussion or by a third researcher. Four evidence quality ratings for mortality, postoperative infection rate, postoperative complications and adverse effects, and venous thromboembolic events (VTE) rate were low, and although the included studies were randomized controlled trials, they were downgraded owing to high risk of bias, wide confidence intervals, and crossing zero lines. The quality of evidence on length of stay was assessed as very low, mainly because some studies had a high risk of bias, high heterogeneity, and high imprecise effect estimates.

### 2.4 Statistical analysis

Stata 14.0 statistical software was used. Odds ratios (RR) and their corresponding 95% confidence intervals were used for binary data, and normalized mean differences (SMD) and their corresponding confidence intervals were used for continuous data. The *I*^2^ statistic and Cochran's *Q*-test were used to estimate heterogeneity. Heterogeneity was categorized as none (0%), low (0%−25%), moderate (25%−50%) and high (>50%). The statistical analysis of heterogeneity tests within forest plots follows distributions with a significance level of α = 0.05. Heterogeneity was detected among studies when *I*^2^ > 50%. Fixed-effects models were employed for pooled effect size analysis in studies without heterogeneity, while random-effects models were utilized in the presence of heterogeneity. When heterogeneity was high (*I*^2^ > 50%), the random-effects model was used because real effect differences were assumed to exist among the included studies. Sensitivity analyses were performed when heterogeneity was present. Subgroup analyses were performed for the primary outcome. Funnel plots were used to detect publication bias. GRADE was used to assess the quality of evidence for each outcome.

## 3 Results

### 3.1 Literature identification

**Figure 1:**
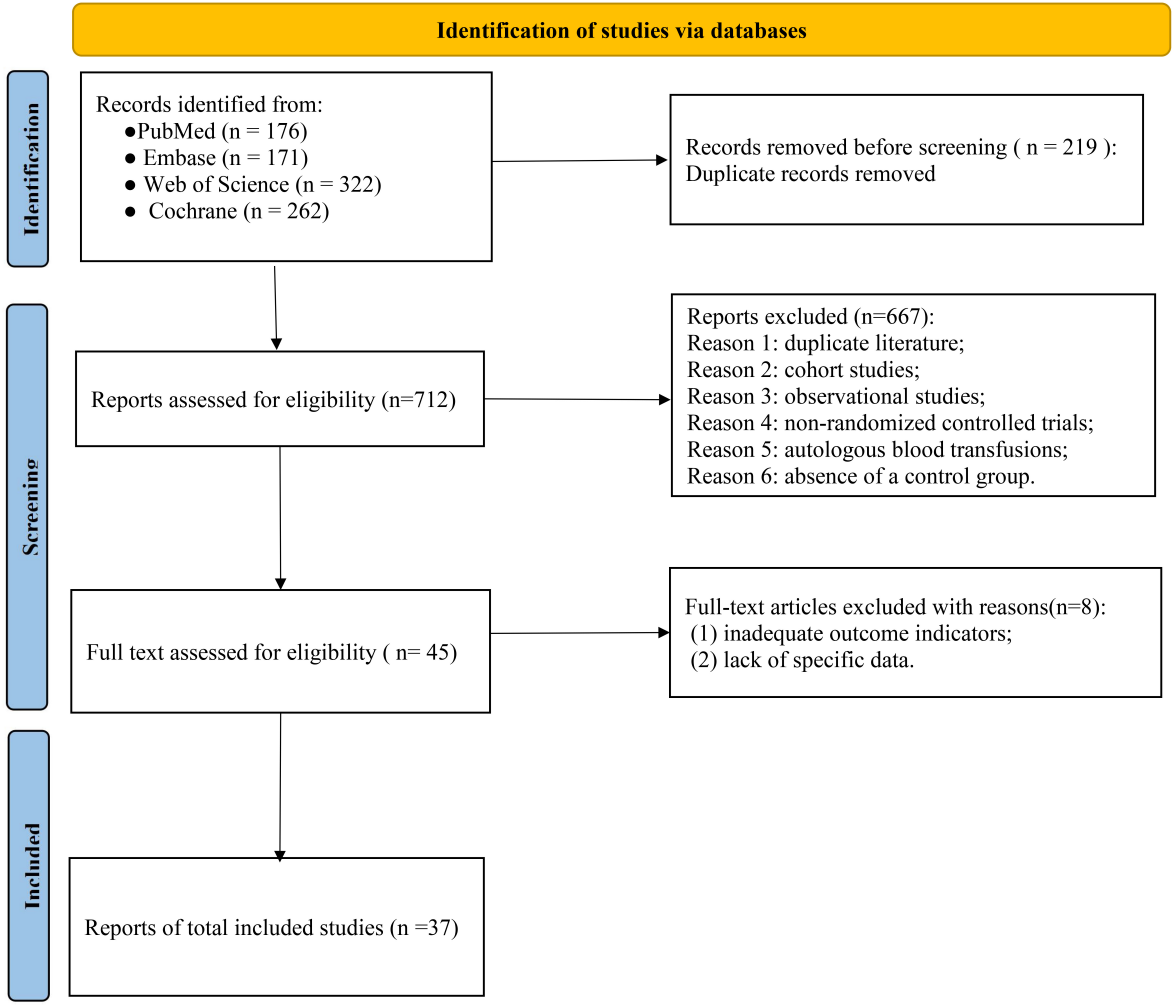
Summary of the literature search strategy.

Out of 931 original studies screened, 74 full-text articles were assessed for eligibility. Upon further review, 37 RCTs met the inclusion criteria and were incorporated into this analysis (Figure 1).

### 3.2 General characteristics

The analysis contained data from 37 randomized controlled studies, with a total of 5,746 patients: 3,215 in the EPO group and 2,531 in the control group. Included studies were of men and/or women aged 32.2–88 years, receiving either low or high doses of EPO by either intravenous (IV) or subcutaneous (SC) routes. Iron supplementation as a concurrent intervention was also reported in some of these studies (Table 1).

**Table 1:** Clinical features of included studies.

**First author**	**Year**	***N***	**Case (T/C)**	**Proportion of males**		**Age (years)**		**EPO Type**	**EPO Dose**	**EPO Duration**	**EPO Route**	**Surgery Type**	**Other Interventions**
				**Test**	**Control**	**Test**	**Control**						
Aydin (7)	2012	92	45/47	0.710	0.700	51 ± 14	56 ± 12	Epoetin β	33,000 U/day	3–4 h before surgery 3 days	IV	Kidney transplant	None
Laupacis (29)	1993	208	77/78	0.510	0.540	64 ± 12	63 ± 12	NR	300 U/kg	Preoperative for 14 days	Subcutaneous	Primary or revision hip arthroplasty	Per os ion (300 mg/day)
			53/78	0.420	0.540	63 ± 15	63 ± 12	NR	300 U/kg	Preoperative for 9 days	Subcutaneous	Primary or revision hip arthroplasty	Per os ion (300 mg/day)
Christodoulakis (24)	2005	204	69/68	0.449	0.412	72.5	70	Epoetin α	150 U/kg/day	Preoperative for 10 days	Subcutaneous	Elective colorectal	Per os iron (200 mg/day) and folic acid (15 mg/day)
			67/68	0.448	0.412	71	70	Epoetin α	300 U/kg/day	Preoperative for 10 days	Subcutaneous	Elective colorectal	Per os iron (200 mg/day) and folic acid (15 mg/day)
D'Ambra (25)	1997	182	63/56	0.570	0.470	62.6 ± 7.8	62.1 ± 8.1	Epoetin α	150 U/kg/day	Daily for 8 days, beginning 5 days before the scheduled operation	Subcutaneous	Elective CABG	Cell saver and per os iron (325 mg/day)
			63/56	0.580	0.470	60.3 ± 7.8	62.1 ± 8.1	Epoetin α	300 U/kg/day	Daily for 8 days, beginning 5 days before the scheduled operation	Subcutaneous	Elective CABG	Cell saver and per os iron (325 mg/day)
Dardashti (10)	2014	70	35/35	0.800	0.770	72.4 ± 8.1	72.5 ± 10.5	NR	400 U/kg	Single dose preoperative	IV	CABG	Cell saver
Dousias (27)	2003	50	23/27	0	0	48.2 ± 4.1	49.2 ± 4.7	NR	600 U/kg/week	Preoperative for 3 weeks	Subcutaneous	Total hysterectomy	Per os iron (200 mg/day)
Dousias (42)	2005	38	20/18	0	0	48.6 ± 7.6	46.9 ± 7.1	NR	200 U/kg/day	Preoperative for 10 days; postoperative for 5 days	Subcutaneous	Radical abdominal or gynecological cancer	Per os iron (200 mg/day)
Faris (18)	1996	185	54/67	0.320	0.260	65 ± 14.7	66 ± 12.2	NR	300 U/kg/day	Preoperative for 10 days; postoperative for 5 days postoperative for 4 days	Subcutaneous	Major orthopedic	Per os iron (325 mg 3 times daily)
			64/67	0.420	0.260	67 ± 13.2	66 ± 12.2	NR	100 U/kg/day	Preoperative for 10 days; postoperative for 5 days postoperative for 4 days	Subcutaneous	Major orthopedic	Per os iron (325 mg 3 times daily)
Feagan (19)	2000	214	46/82	0.114	0.128	67.3 ± 11.0	67.8 ± 11.9	Epoetin α	40,000 U/week	Preoperative for 4 weeks	Subcutaneous	Primary hip arthroplasty	Per os iron (400 mg/day)
			86/82	0.076	0.128	68.9 ± 10.8	67.8 ± 11.9	Epoetin α	20,000 U/week	Preoperative for 4 weeks	Subcutaneous	Primary hip arthroplasty	Per os iron (400 mg/day)
Gaston (12)	2006	50	25/25	1.0	1.0	58	60	Epoetin α	40,000 U/week	Preoperative for 2 weeks	NR	Radical prostatectomy	None
Haljan (43)	2009	91	18/23	1.0	1.0	60	62	NR	1,500 U/kg	Preoperative in 3 divided doses	IV	CABG	None
			26/23	1.0	1.0	57	62	NR	750 U/kg	Preoperative in 3 divided doses	IV	CABG	None
			24/23	1.0	1.0	61	62	NR	375 U/kg	Preoperative in 3 divided doses	IV	CABG	None
Heiss (44)	1996	27	17/10	0.412	0.200	66	61	NR	150 U/kg	Preoperative for 10 days, postoperative for 2 days	Subcutaneous	Colorectal surgery	Per os iron (200 mg/day) and folic acid (5 mg/day)
Kateros (45)	2010	79	38/41	0.237	0.293	78	77.6	Epoetin α	20,000 U/day	10 days from admission	NR	Intertrochanteric fracture repair	Per os iron (100 mg/day)
Kettelhack (21)	1998	102	48/54	0.438	0.407	71	67	Epoetin β	20,000 U/day	Preoperative for 5 days; postoperative for 4 days	Subcutaneous	Right hemicolectomy	Per os or IV iron
Kim (26)	2013	98	49/49	0.510	0.512	63	62	Epoetin α	300 U/kg	After induction	IV	Complex cardiac valvular	Cell saver
Kim (9)	2016	60	31/29	0.610	0.410	64	65	NR	500 U/kg	10 min before incision	IV	Various cardiac	Cell saver
Kosmadakis (30)	2003	63	31/32	0.484	0.594	67.1	66.4	Epoetin α	300 U/kg/day	Preoperative for 7 days; postoperative for 6 days	Subcutaneous	Nonmetastatic gastrointestinal cancer surgery	IV iron (100 mg/day)
Luchette (46)	2012	192	97/95	0.670	0.620	32.2	33.5	Epoetin α	10,000–40,000 U	Once preoperative based on hemoglobin level	Subcutaneous	Major blunt orthopedic trauma	Per os iron
Na (23)	2011	108	54/54	NR	NR	69.4	69.4	rHuEPO-β	3,000 U/day	Once preoperative, postoperative for 2 days	Subcutaneous	Bilateral total knee arthroplasty	Erythropoietin only: IV iron (200 mg/day)
Norager (22)	2006	151	75/76	0.560	0.618	65	63	Darbepoetin	150–300 μg/week	Preoperative for 10 days; postoperative for 15 days	Subcutaneous	Colorectal	Per os iron (200 mg/day), folic acid (5 mg/day) + vitamin B12 injections
Qvist (47)	1999	81	38/43	0.316	0.465	69	69	NR	150–300 U/kg/day	Preoperative 300 U/kg for 4 days, postoperative 150 U/kg for 7 days	Subcutaneous	Elective colorectal	Per os iron (200 mg/day)
Sowade (15, 16)	1997	72	36/36	0.778	0.778	54.3	57	Epoetin β	5,000 U/kg	Preoperative 5 doses given	IV	Cardiac	Per os iron (300 mg/day)
Weber (20)	2005	704	467/237	0.101	0.105	67	66.7	Epoetin α	40,000 U/week	Preoperative for 3 weeks	Subcutaneous	Gastrectomy	IV iron (40 mg/day)
Weltert (31)	2010	320	158/162	0.840	0.830	66.9	66.4	NR	40,000 U/week	Preoperative for 3 weeks	Subcutaneous	Elective major orthopedic	Per os iron
Weltert (48)	2015	600	300/300	0.750	0.730	75	74	NR	8,000–14,000 U	Preoperative 14,000 U once, postoperative 8,000 U for 2 days	Subcutaneous	CABG	None
Wurnig (14)	2001	175	65/51	0.200	0.383	62.5	62	Epoetin β	125 IU/kg	Preoperative for 3–4 weeks	Subcutaneous	Elective surgery (orthopedic surgery and cardiac surgery)	Per os iron (200–300 mg/day)
			59/51	0.313	0.383	66	62	Epoetin β	250 IU/kg	Preoperative for 3–4 weeks	Subcutaneous	Elective surgery (orthopedic surgery and cardiac surgery)	Per os iron (200–300 mg/day)
Yoo (49)	2011	74	37/37	0.378	0.351	56	59	NR	500 U/kg	Once before surgery	IV	Cardiac valve surgery	IV iron (200 mg) once
Urena (11)	2017	100	48/52	0.458	0.519	81 ± 7	81 ± 7	Darbepoetin	0.75 μg/kg	The days 10 (±4) and 1 (±1) prior	Subcutaneous	Undergoing transcatheter aortic valve implantation	Iron
Totonchi (50)	2022	66	33/33	0.394	0.333	59.4 ± 8.9	61.4 ± 9.3	NR	500 IU/kg/day	Surgery and 3 days afterward	Subcutaneous	Cardiac surgery	Intravenous ferric carboxymaltose a day before surgery
Saour (51)	2024	123	62/61	0.440	0.440	70.5 ± 9.0	69.7 ± 8.8	NR	40,000 IU	The day before surgery or at ICU admission	Subcutaneous	Cardiac surgery	20 mg/kg intravenous ferric carboxymaltose
So-Osman (36)	2014	683	339/344	0.120	0.130	71 ± 12	71 ± 12	NR	40,000 U	Days—21, 14, 7, and on the day of surgery (day 0)	Subcutaneous	Elective total hip- and knee-replacement surgery	Ferrofumarate 200 mg three times per day (195 mg Fe2+ a day) for 3 weeks before surgery
Kong (28)	2022	156	79/77	0.481	0.519	75	73	Darbepoetin	200 μg	On the day of randomization	Subcutaneous	Cardiac surgery	Ferric derisomaltose (previously known as iron isomaltoside, Monofer^^®^^) 1,000 mg (or 20 mg kg weighed less than 50 kg) by intravenous infusion over 60 min
Hayashi (17)	1994	86	28/28	0.571	0.571	56.9 ± 7.8	57.4 ± 8.0	NR	12,000 IU	Days 21, 14, and 7	Subcutaneous	Cardiac surgery	200 mg per day were given for 21 days before operation
			30/28	0.733	0.571	61.0 ± 6.4	57.4 ± 8.0	NR	24,000 IU	Days 21, 14, and 7	Subcutaneous	Cardiac surgery	200 mg per day were given for 21 days before operation
Yazicioglu (52)	2001	53	25/28	NR	NR	NR	NR	NR	100 IU/kg	4 days before the operation	IV	Cardiac surgery	400 ml of their own blood on the morning of the operation
Loo (53)	1996	29	14/15	0.643	0.733	47.4 ± 17.0	47.8 ± 11.7	NR	Starting dose was 150 U/kg, increased at weekly intervals by 30 U/kg/week	Three doses per week, for a maximum period of 12 weeks	Subcutaneous	Kidney transplantation	Iron substitution (Ferogradumet CR, iron sulfate, Abbott) was administered at a peroral dosage of 525 mg/day
Cao (8)	2020	102	35/32	0.171	0.125	67.77 ± 8.45	69.07 ± 6.46	NR	10,000 IU	8 daily doses of 10,000 IU,3 days before surgery	Subcutaneous	Total knee arthroplasty	3 daily doses of 200 mg intravenous iron sucrose
			35/32	0.143	0.125	67.43 ± 8.34	69.07 ± 6.46	NR	10,000 IU	The day of surgery	Subcutaneous	Total knee arthroplasty	3 daily doses of 200 mg intravenous iron sucrose

N, number of patients; T/C, test/control; NR, no report; CABG, coronary artery bypass graft; IV, intravenous injection; EPO, erythropoieti.

Table 1 presents the study characteristics, including the total participant count, distribution between intervention and control groups, age range, proportion of male patients, erythropoietin dosage and administration route, surgery type, and other blood conservation strategies. The studies encompassed a range of 29–704 cases, totaling 5,746 patients across 37 publications, with an average sample size of 155. The age of patients ranged from 32.2 to 88 years, with a higher proportion of women than men. Further details on the specific interventions in each group are also listed in Table 1.

### 3.3 Risk of bias of the included studies

**Figure 2:**
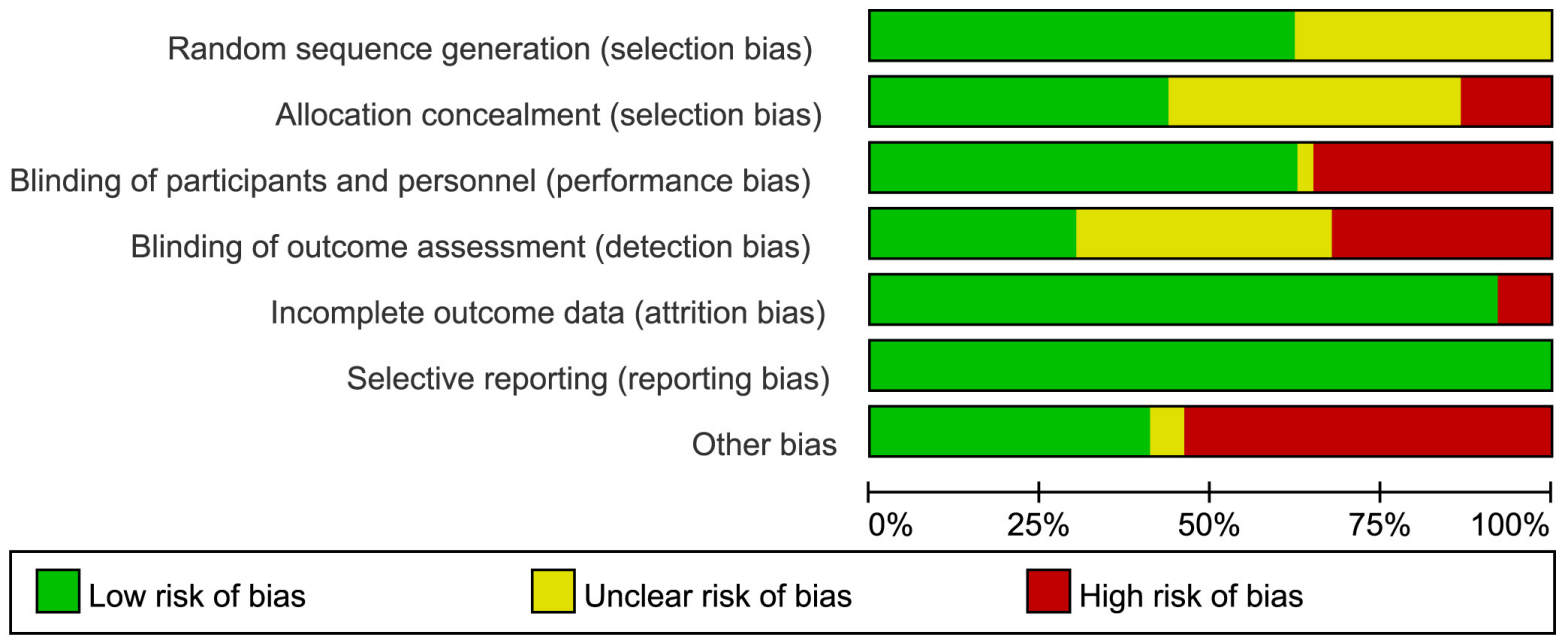
Risk of bias graph: review authors' judgements about each risk of bias item presented as percentages across all included studies.

**Figure 3:**
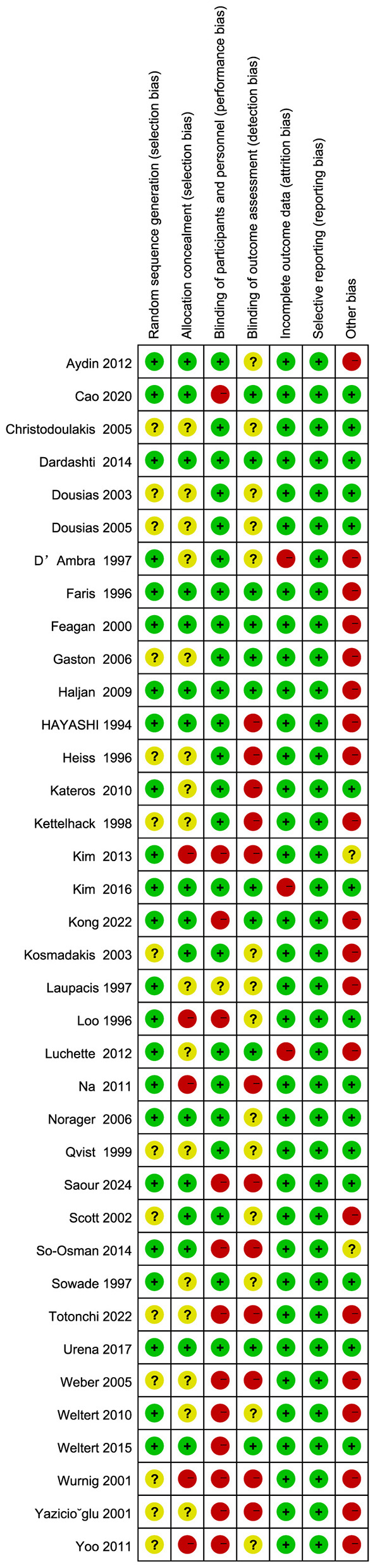
Risk of bias summary review authors' judgements about each risk of bias item for each included study.

We used the Cochrane Risk of Bias Assessment Tool to evaluate the methodological quality of 37 studies [Aydin et al. (7), Cao et al. (8), Kim et al. (9)]. Figure 2 presents the results of the quality assessment. Two studies [Dardashti et al. (10), Urena et al. (11)] had low risk of overall bias with respect to selection bias (random sequence generation), implementation bias (blinding participants and investigators), outcome assessment blinding, loss to follow-up bias (incomplete outcome data), and other sources of bias (Figure 3). Risk of bias over 50% of the studies showed low risk for randomization sequence generation and blinding. Low risk of bias in allocation concealment, outcome assessment blinding, and other sources of bias affected less than 50% Selective Reporting. All studies were rated as at low risk of selective reporting bias. Two studies [Dardashti et al. (10), Urena et al. (11)] were rated as low risk across all categories of domains: random sequence generation, allocation concealment, blinding of participant and personnel, blinding of outcome assessment, incomplete outcome data, selective reporting, and other bias.

### 3.4 Assessment of quality of evidence by GRADE

We evaluated the quality of evidence for both primary and secondary outcome measures in accordance with GRADE, and summarized the results in Supplementary Table S2.

For the primary outcome, i.e., perioperative allogeneic transfusion rate, the quality of evidence was assessed as low. Although most of the included studies were randomized controlled trials, they were downgraded owing to limitations and inconsistencies resulting in high risk of bias and high heterogeneity.

For secondary outcome measures, the quality of evidence for the number of allogenic red blood cell fusions was rated as low, mainly due to the high risk of bias in some studies and high heterogeneity.

### 3.5 Effects of interventions

We compared the included studies for the effects of erythropoietin intervention and placebo or the absence of erythropoietin.

### 3.6 Primary outcome

**Figure 4:**
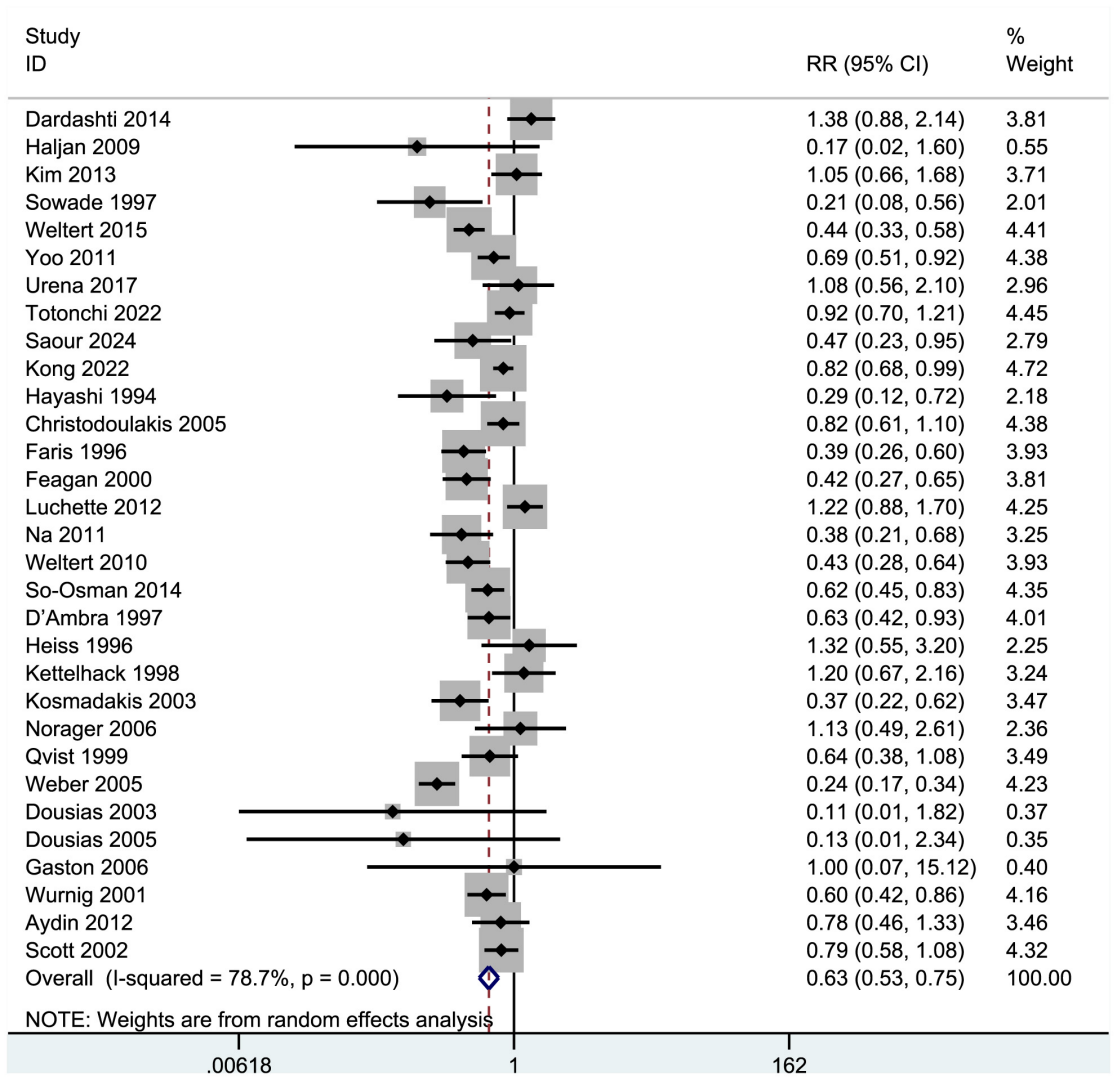
Forest plot of blood transfusion rate.

Thirty-one studies examined patients receiving allogeneic red blood cell transfusions during the peri-operative period, with at least one unit transfused (Figure 4). These trials encompassed 5,119 participants: 2,839 (55.5%) were assigned to a group receiving human erythropoietin with or without iron, while 2280 (44.5%) served as controls.

**Figure 5:**
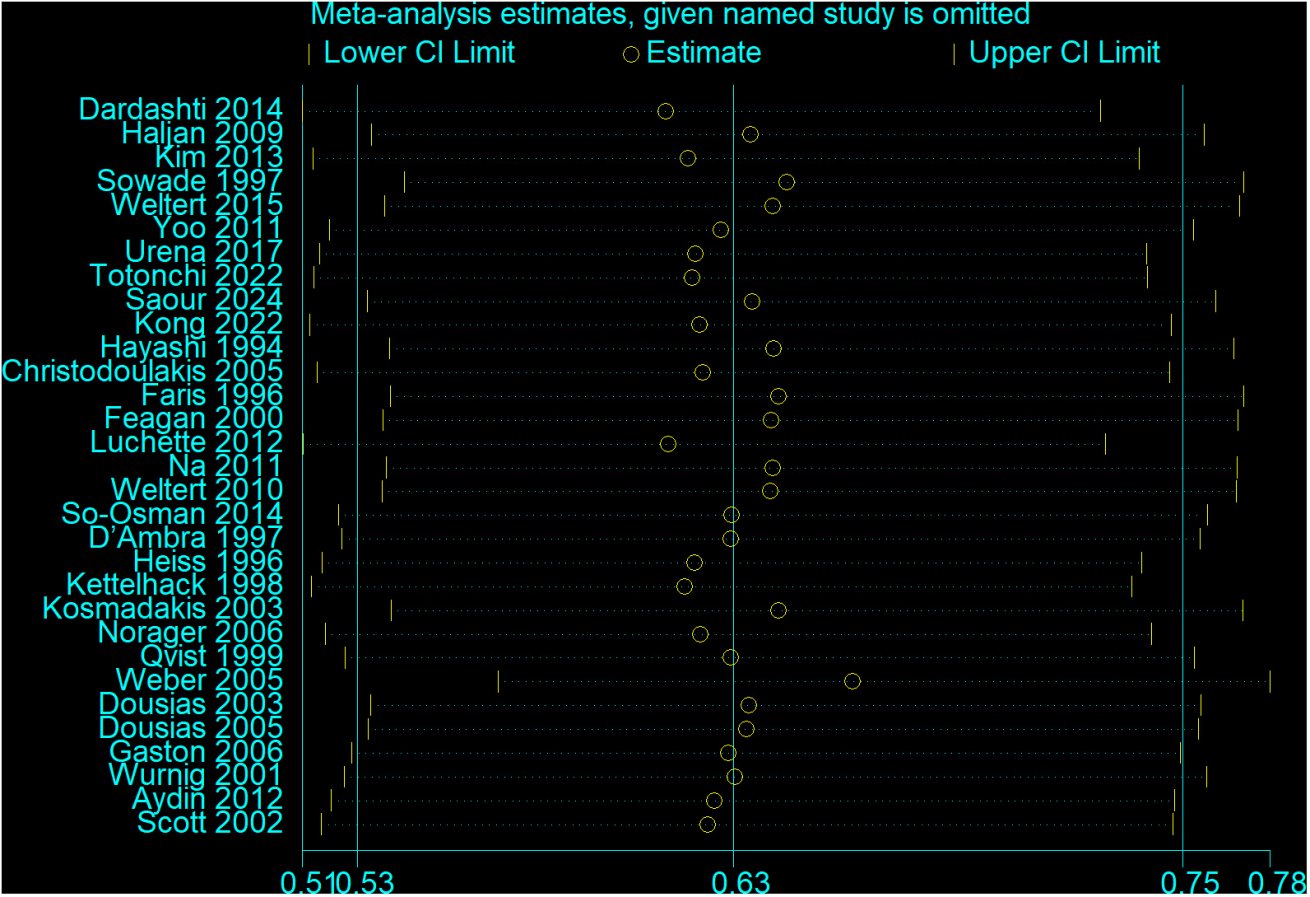
Sensitivity analysis of blood transfusion rate.

Results indicated that recombinant human erythropoietin, with or without iron, significantly reduced the need for allogeneic transfusions in patients (RR = 0.63, 95% CI = 0.53–0.75, *p* = 0.000; *I*^2^ = 78.7%; see Figure 4). A meta-analysis of these 31 RCTs confirmed a significant reduction in transfusion risk (RR = 0.63, 95% CI: 0.53–0.75), although high heterogeneity (*I*^2^ = 78.7%) suggested variability in effect based on patient type and intervention protocol. The significant heterogeneity among the selected studies warranted further exploration. Consequently, we performed a sensitivity analysis (Figure 5), whose results indicated that excluding any single study maintained the pooled effect size (RR) between 0.51 and 0.78, with confidence intervals consistently below 1.0, demonstrating robust results.

### 3.7 Subgroup analysis

Erythropoietin administration demonstrated a significant reduction in allogeneic transfusion rates among perioperative patients relative to controls. In the subgroup analyses, we tested types of surgery and EPO subtypes. Specifically, subgroup analysis based on surgical type revealed a significant decrease in transfusion rates for cardiac surgery (RR = 0.68, 95% CI = 0.51–0.91, *p* = 0.000, *I*^2^ = 78.8%), while EPO administration in orthopedic surgery also led to a significant reduction in transfusions (RR = 0.57, 95% CI = 0.41–0.80, *p* = 0.000, *I*^2^ = 82.3%). Further subgroup analysis focusing on EPO molecular variants indicated that EPO-α significantly lowered transfusion rates (RR = 0.65, 95% CI = 0.45–0.95, *p* = 0.000, *I*^2^ = 86.9%), with EPO-β also demonstrating a significant reduction in transfusions (RR = 0.58, 95% CI = 0.37–0.92, *p* = 0.010, *I*^2^ = 69.9%).

#### 3.7.1 Subgroup analysis of surgery types

**Figure 6:**
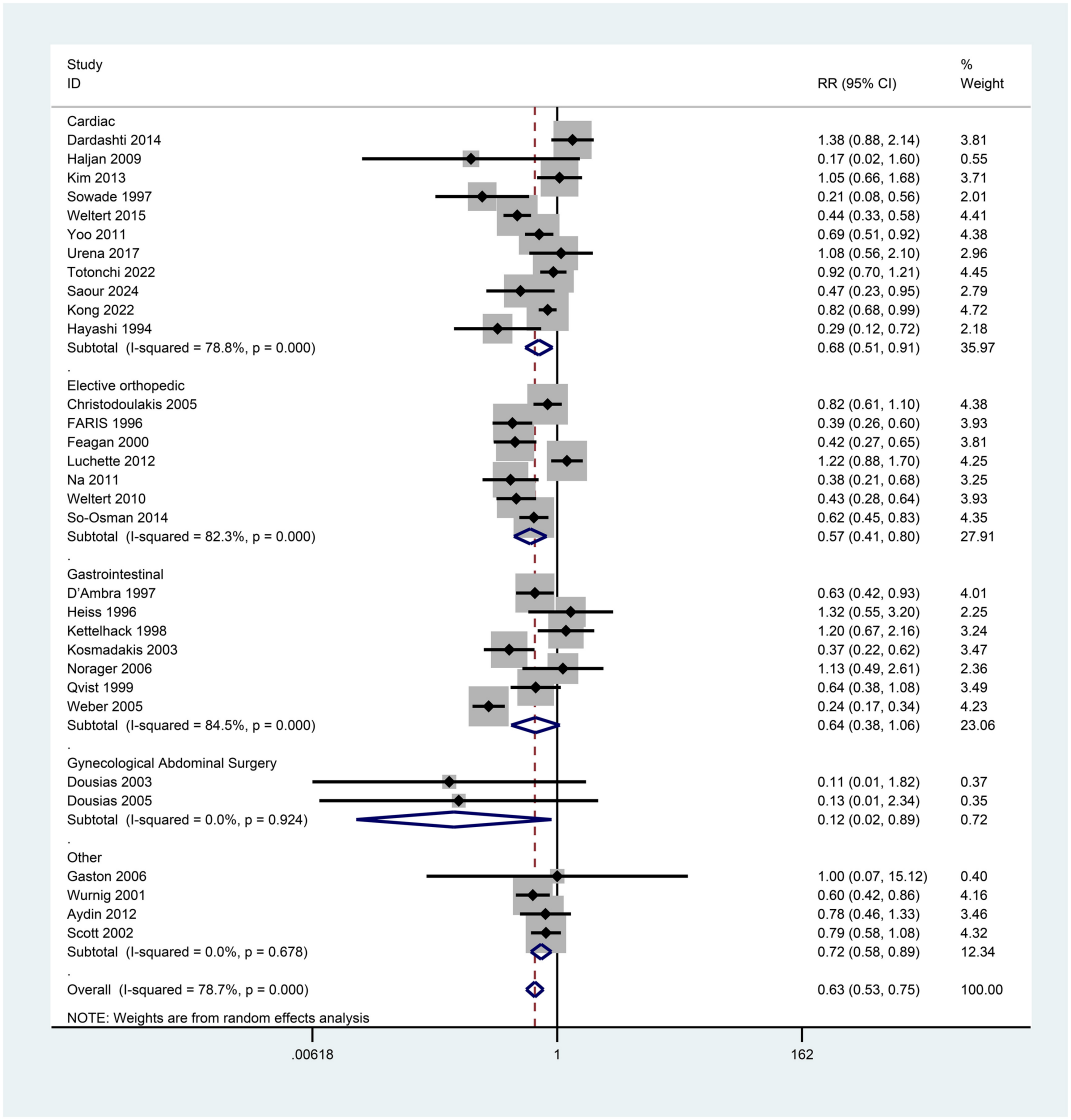
Forest plot of subgroup analysis for transfusion rates by surgical category.

In delineating patient characteristics, we identified 31 studies examining primary outcome indicators. Specifically, 11 studies pertained to cardiac surgery, seven to orthopedic surgery, seven to gastrointestinal surgery, and two to gynecological abdominal surgery, with the remaining four studies encompassing diverse procedures (Aydin et al. (7) on kidney transplantation, Gaston et al. (12) on radical prostatectomy, Scott et al. (13) on head and neck tumor resection, and Wurnig et al. (14) on elective orthopedic and cardiac surgeries). Subsequently, we constructed a forest plot for subgroup analysis across various surgical categories (Figure 6). The results indicated that cardiac surgery accounted for 35.97% of cases, with a pooled relative risk (RR) of 0.68 (95% CI: 0.51–0.91), suggesting a 32% reduction in blood transfusion rates. Heterogeneity was high (*I*^2^ = 78.8%, *p* < 0.001), consistent with the overall heterogeneity. Notably, Sowade et al. (15, 16) (RR = 0.21) and Hayashi et al. (17) (RR = 0.29) demonstrated the most substantial effects. In contrast, Dardashti et al. (10) (RR = 1.38) and Urena et al. (11) (RR = 1.08) indicated ineffectiveness or a possible increase in risk, although their confidence intervals included 1. For orthopedic surgery, accounting for 27.91%, the pooled RR was 0.57 (95% CI: 0.41–0.80), indicating a 43% reduction in transfusion rates. Heterogeneity remained high (*I*^2^ = 82.3%, *p* = 0.000). Faris et al. (18) (RR = 0.39) and Feagan et al. (19) (RR = 0.42) exhibited significant effects, while Luchette et al. (46) (RR = 1.22) suggested a potential increase in risk, with its confidence interval also crossing 1. Gastrointestinal surgery represented 22.6% of the cases analyzed. The combined relative risk (RR) was 0.64 (95% CI = 0.38–1.06), signifying a notable 36% decrease in the incidence of blood transfusions, albeit with a confidence interval spanning 1 (hence not statistically significant). The observed heterogeneity was substantial (*I*^2^ = 84.5%, *p* = 0.000). Notably, Weber et al. (20) (RR = 0.24) demonstrated the most pronounced effect, while Kettelhack et al. (21) (RR = 1.20) and Norager et al. (22) (RR = 1.13) suggested lack of efficacy in this context. Gynecological surgeries accounted for 6.4% of the cases. The pooled RR was 0.12 (95% CI = 0.02–0.89), resulting in a significant (88%) reduction in blood transfusion rates, although the limited sample size (only two studies) warrants caution. It is advisable to infer that the current evidence favors a notable decrease in transfusion rates for gynecological surgeries. However, the reliability of this conclusion warrants validation through additional high-quality studies, given the limitations in sample size. Heterogeneity was minimal (*I*^2^ = 0%), indicating high result consistency. Other surgical procedures comprised 12.34% of the cases. The combined RR was 0.72 (95% CI = 0.58–0.89), correlating with a noteworthy (28%) decrease in blood transfusion rates. Gaston et al. (12) (RR = 1.00) exhibited a wide confidence interval (0.07–15.12), reflecting considerable uncertainty in the findings. Conversely, Wurnig et al. (14) (RR = 0.60) demonstrated a significant effect.

#### 3.7.2 Subgroup analysis of molecular types

**Figure 7:**
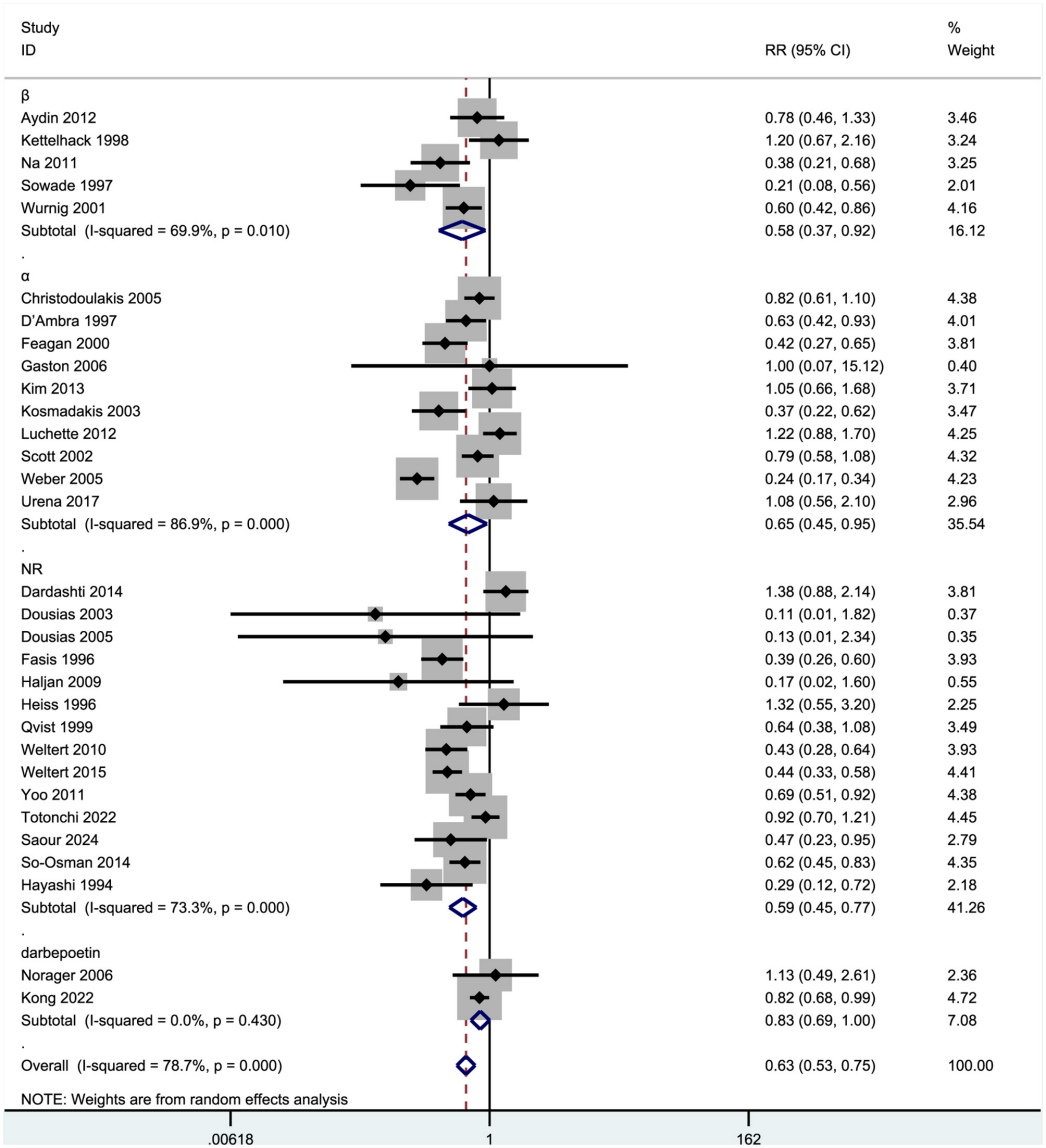
Forest plot of subgroup analysis for transfusion rates by molecular types.

A review of the literature revealed that erythropoietin can be categorized into three molecular types: first-generation short-acting (e.g., epoetin alfa), second-generation long-acting (e.g., darbepoetin alfa), and third-generation fusion protein (e.g., methoxy PEG-epoetin beta). Accordingly, we categorized the 31 studies included in our analysis into three groups based on molecular type: EPO-α, EPO-β, and darbepoetin (Figure 7).

Subgroup analysis based on molecular type revealed that the β subgroup (representing conventional EPO-β) comprised five studies [Aydin et al. (7), Kettelhack et al. (21), Na et al. (23), Sowade et al. (15, 16), Wurnig et al. (14)], demonstrating a pooled RR of 0.58 (95% CI: 0.37–0.92), leading to a significant 42% reduction in transfusion rates (*p* < 0.05). However, notable heterogeneity was observed within this subgroup (*I*^2^ = 69.9%, *p* = 0.010). Particularly, Na et al. (23) (RR = 0.38) and Sowade et al. (15, 16) (RR = 0.21) exhibited the most pronounced effects, with their confidence intervals excluding 1. Conversely, Aydin et al. (7) (RR = 0.78) and Kettelhack et al. (21) (RR = 1.20) did not show significant effects. In contrast, the α subgroup (representing conventional EPO-α) encompassed 10 studies [e.g., Christodoulakis et al. (24), D'Ambra et al. (25), and Feagan et al. (19)] with a combined RR of 0.65 (95% CI: 0.45–0.95), with a significant 35% decrease in transfusion rates (*p* < 0.05). Nevertheless, a remarkably high level of heterogeneity was detected within this subgroup (*I*^2^ = 86.9%, *p* < 0.001). Specifically, Weber et al. (20) (RR = 0.24) and Kosmadakis et al. (30) (RR = 0.37) demonstrated significant effects. In contrast, Gaston et al. (12) (RR = 1.00) exhibited an exceedingly wide confidence interval (0.07–15.12), rendering the result unreliable. The group comprising 14 studies with unspecified molecular types demonstrated a pooled relative risk (RR) of 0.59 (95% CI: 0.45–0.77), leading to a significant 41% reduction in transfusion rates (*p* < 0.001), albeit with substantial heterogeneity (*I*^2^ = 73.3%, *p* < 0.001). Notably, seven studies, including Faris et al. (18) (RR = 0.39) and Weltert et al. (31) (RR = 0.43), exhibited significant effects, while Dousias et al. (27) (RR = 0.11) reported unstable results characterized by a wide confidence interval (0.01–1.82). Within the darbepoetin (a long-acting erythropoietin analog) subgroup, consisting of two studies [Norager et al. (22), Kong et al. (28)], the pooled RR was 0.83 (95% CI: 0.69–1.00), indicating a 17% reduction in transfusion rates, although statistical significance was not achieved (CI includes 1). This subgroup demonstrated no heterogeneity (*I*^2^ = 0%), suggesting a more uniform effect compared with other subgroups. The observed weaker effect size in the darbepoetin subgroup may be attributed to variances in pharmacokinetics or suboptimal dose adjustments.

### 3.8 Publication bias

**Figure 8:**
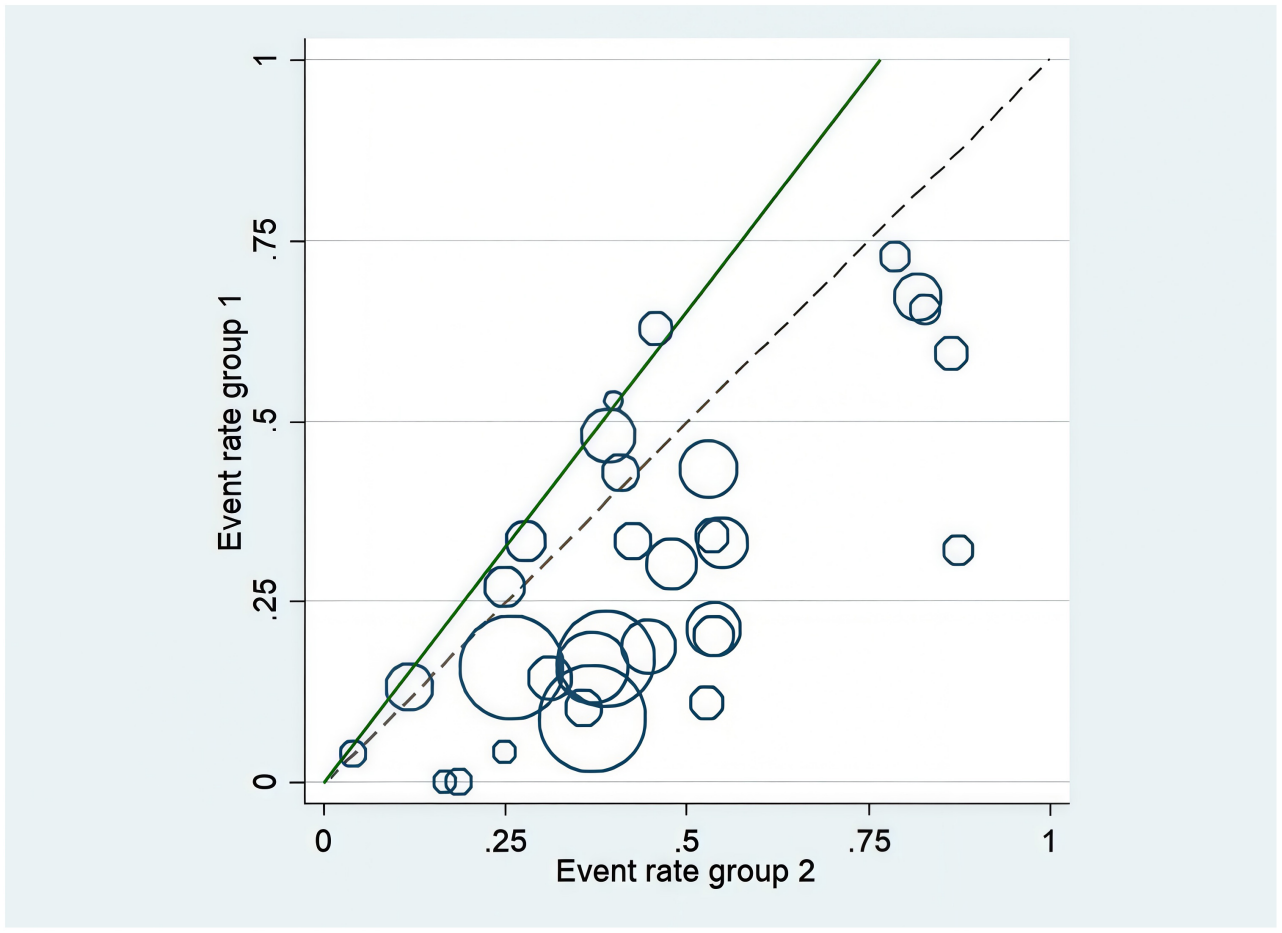
L'Abbé plot of blood transfusion rate.

**Figure 9:**
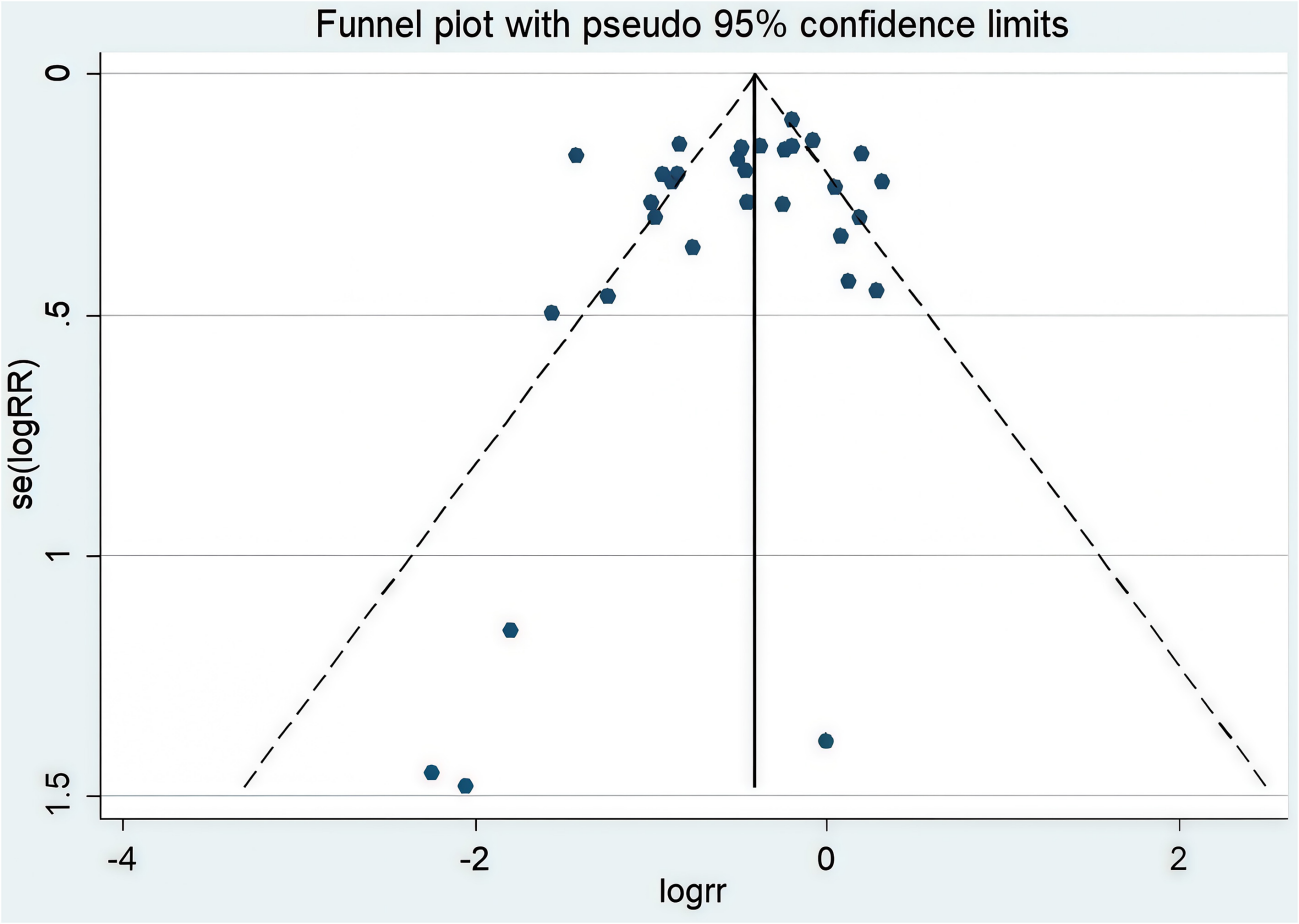
Funnel plot of blood transfusion rate.

**Figure 10:**
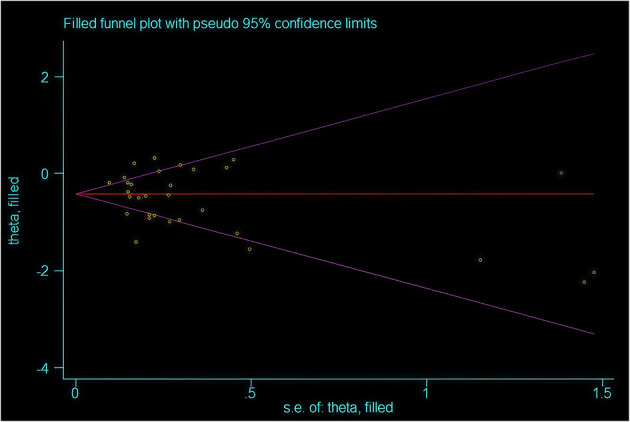
Funnel plot after trimming and filling.

L'Abbe Plot and funnel plots were drawn to examine whether there was publication bias in this study. The L'Abbe plot, although lacking scatter points, suggested—based on the axes structure (with the *Y*-axis representing the event rate in the EPO group) and sensitivity analysis—that points likely concentrated below the diagonal, implying a reduced blood transfusion rate in the intervention group (see Figure 8). Funnel plot symmetry indicated that there is no publication bias. The funnel plot exhibited asymmetry, with potential small-sample studies missing at the bottom (no points on the right and unshown on the left), indicating possible non-publication bias of negative results (see Figure 9). The adjusted effect size, corrected for bias using the trim-and-fill method, is depicted in Figure 10.

To further correct for publication bias, we created a clipped funnel plot. After correcting for publication bias, the evidence continues to support the intervention's ability to reduce blood transfusion rates, although the effect size should be adjusted from RR = 0.53 to RR = 0.63 (see Figure 10). It is advisable to consider these benefits within clinical contexts, such as prioritizing high-risk patients. The conclusion remains valid following bias correction with the trim-and-fill method.

### 3.9 Secondary outcomes

#### 3.9.1 The number of allogeneic red blood cell transfusions (units/patient)

**Figure 11:**
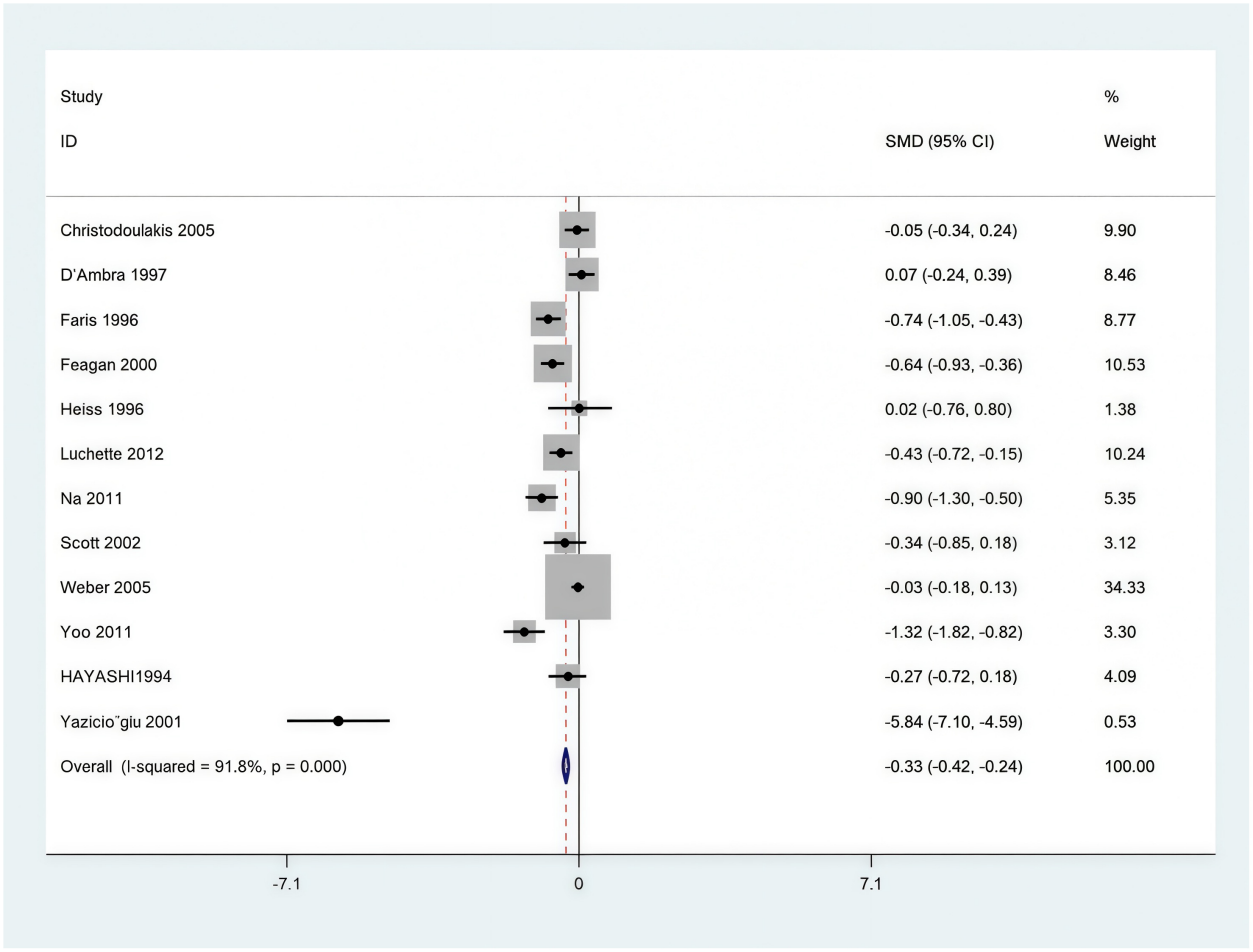
Allogeneic red blood cell transfusion volume (units per patient).

Twelve studies [Christodoulakis et al. (24), D'Ambra et al. (25), Faris et al. (18)], examined the red blood cell units transfused per individual in EPO intervention groups. Effect size showed standardized mean difference (SMD) = −0.33 (95% CI: −0.42 to −0.24); Significance showed the confidence interval excludes (*p* < 0.001); Effect direction showed that the negative SMD signifies a significant reduction in blood transfusion volume with EPO treatment; Variability showed that substantial heterogeneity was observed (*I*^2^ = 91.8%, *p* < 0.001), indicating considerable diversity across studies. In conclusion, the erythropoietin (EPO) treatment group (whether combined with iron supplementation or not) demonstrated a significant difference in the number of red blood cell transfusion units compared with the control group (see Figure 11). EPO administration significantly reduced the blood transfusion volume (SMD = −0.33).

#### 3.9.2 Mortality

**Figure 12:**
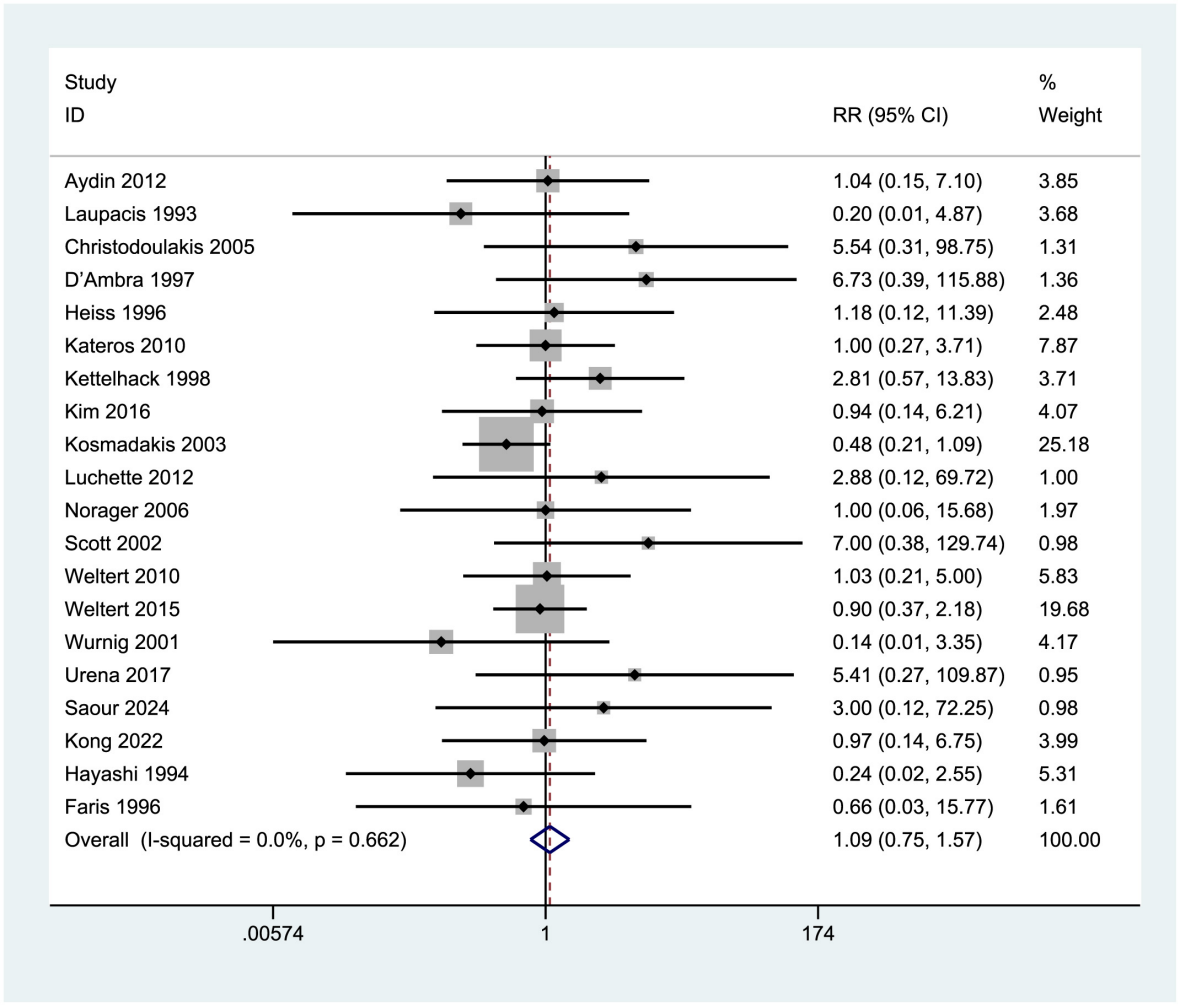
Forest plot of mortality.

Twenty studies [Aydin et al. (7), Laupacis et al. (29), Christodoulakis et al. (24)] with a total of 3,122 participants assessed 30-day postoperative mortality rates. The combined RR was 1.09 (95% CI = 0.75–1.57), indicating no significant difference (*p* > 0.05) as the confidence interval spanned 1 (0.75–1.57). Heterogeneity was minimal (*I*^2^ = 0%, *p* = 0.662), as depicted in Figure 12.

#### 3.9.3 Postoperative infection rate

**Figure 13:**
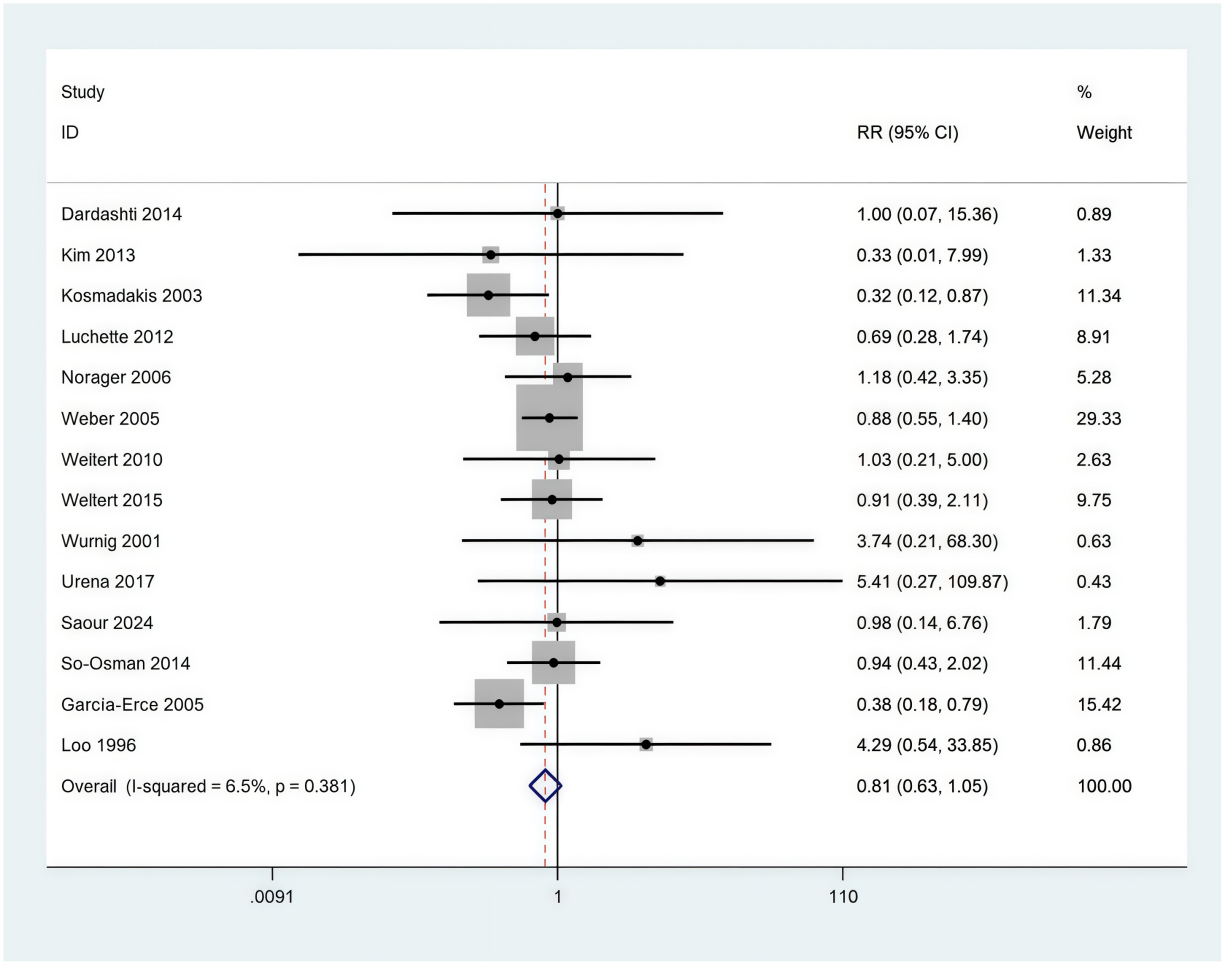
Forest plot of postoperative infection rates.

Thirteen studies [Dardashti et al. (10), Kim et al. (26), Kosmadakis et al. (30)] comprising 3,298 subjects were analyzed to assess postoperative infection rates. The forest plot (Figure 13) analysis revealed no significant difference in postoperative infection incidence between the compared groups. The pooled RR was 0.81 (95% CI: 0.63–1.05), indicating a 19% reduction in infection risk in the EPO intervention group. The 95% CI of 0.63–1.05 included 1, suggesting non-significance (*p* > 0.05). Heterogeneity was notably low (*I*^2^ = 6.5%, *p* = 0.381), indicating high consistency among study outcomes.

#### 3.9.4 Postoperative complications and adverse effects

**Figure 14:**
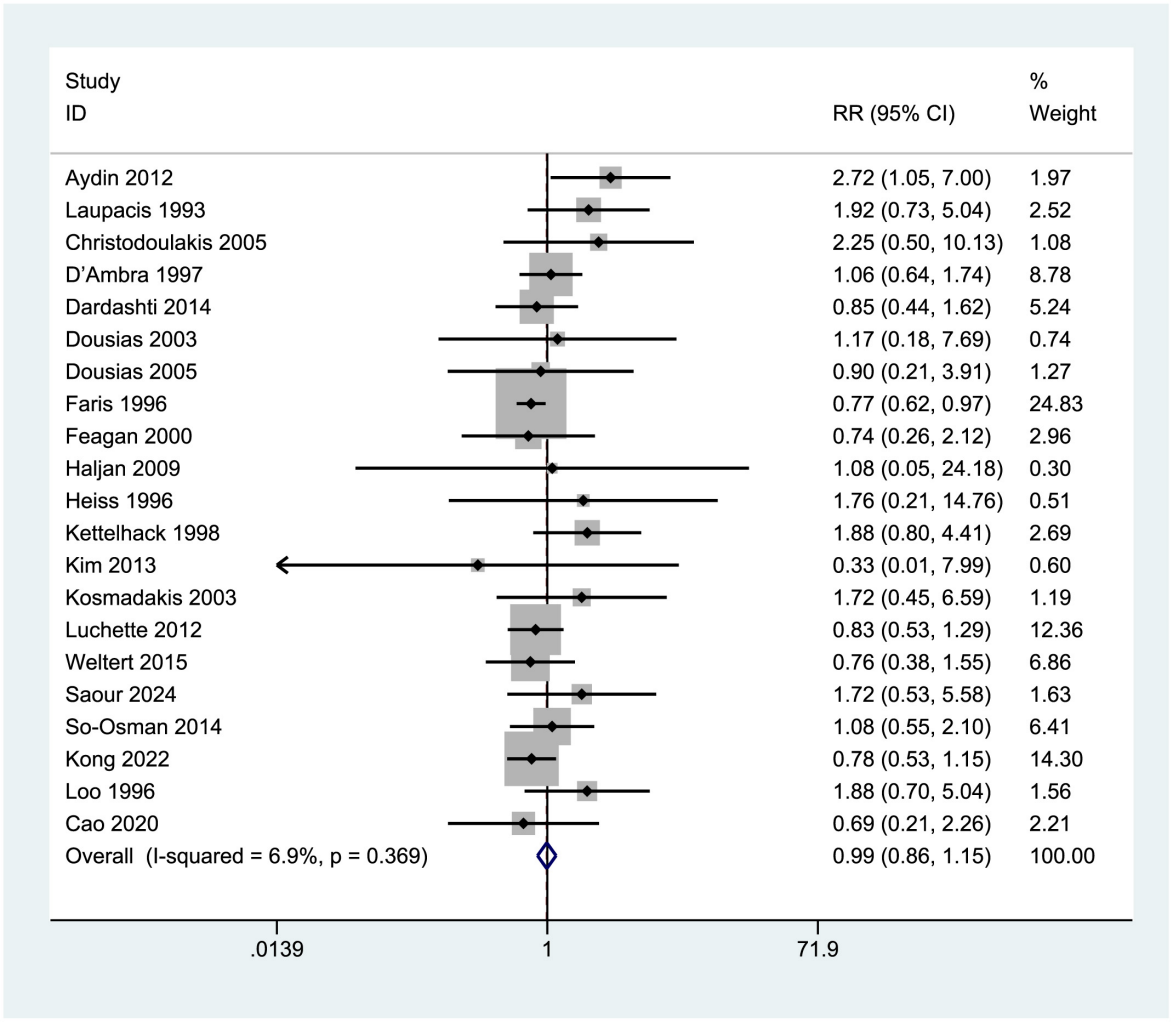
Incidence rates of postoperative complications and adverse reactions.

Twenty-one studies [Aydin et al. (7), Laupacis et al. (29), Christodoulakis et al. (24)] were included in the analysis to assess postoperative complications and adverse reactions. The forest plot results (Figure 14) indicated no significant difference between the two groups in the incidence of postoperative complications and adverse reactions. The pooled RR was 0.99 (95% CI: 0.86–1.15), with extremely low heterogeneity (*I*^2^ = 6.9%, *p* = 0.369), as the confidence interval (0.86–1.15) encompassed 1.0 (*p* > 0.05). The results were not statistically significant (*p* > 0.05).

#### 3.9.5 Venous thromboembolic events (VTE) rate [deep vein thrombosis (DVT)/pulmonary embolism (PE)]

**Figure 15:**
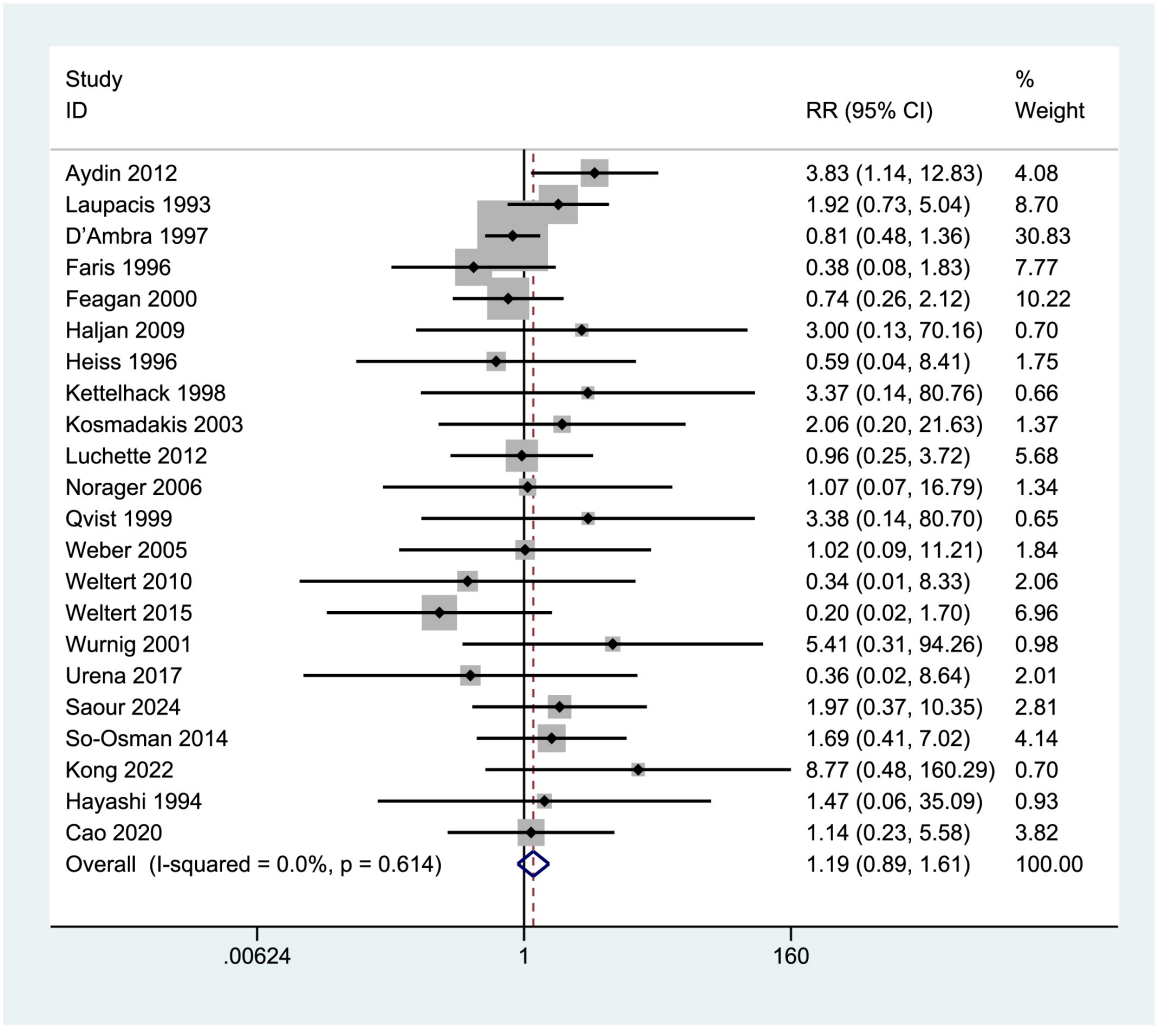
Forest plot of venous thromboembolism events (VTE) rate.

Twenty-two studies [Aydin et al. (7), Laupacis et al. (29), D'Ambra et al. (25), Cao et al. (8)] were analyzed to assess postoperative complications and adverse reactions, involving a total of 4,508 participants. The forest plot results (see Figure 15) indicated no significant difference in the occurrence of postoperative complications and adverse reactions between the two cohorts. The combined RR was 1.19 (0.89, 1.61), with minimal heterogeneity (*I*^2^ = 0.000%, *p* = 0.528). The confidence interval spanned 1 (0.84, 1.61), signifying a lack of statistical significance (*p* > 0.05).

#### 3.9.6 Hospital length of stay (days)

**Figure 16:**
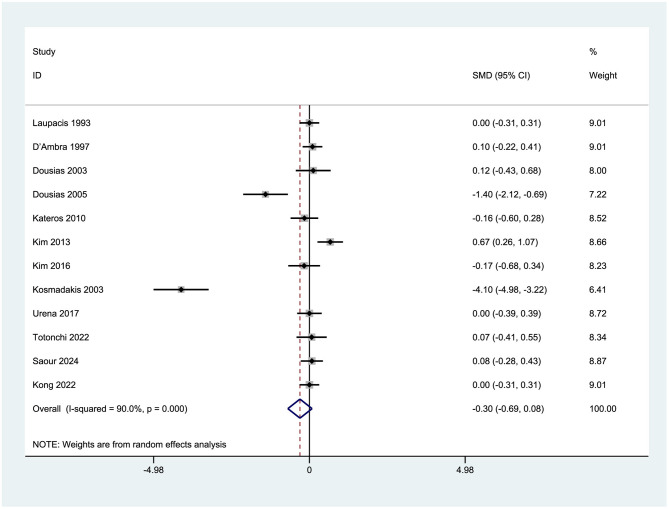
Forest plot of hospital length of stay (days).

Thirteen clinical trials, comprising 1,171 subjects, were examined to assess the impact of erythropoietin administration on hospital length of stay [Laupacis et al. (29), D'Ambra et al. (25), Dousias et al. (27)]. The pooled effect size (ES) was −0.30 (95% CI: −0.69 to 0.08), indicating a non-significant reduction in the average hospital stay duration by approximately 7.2 h in the intervention group (converted from days to hours). The inclusion of within the 95% confidence interval suggests a lack of statistical significance (*p* > 0.05), implying that EPO administration does not lead to a routine reduction in hospital stay duration. The high heterogeneity observed (*I*^2^ = 90.0%) indicates substantial influence from confounding variables on the results (see Figure 16).

## 4 Discussion

This article includes findings from 37 RCTs indicating a significant reduction in perioperative allogeneic transfusion associated with preoperative erythropoietin administration, the primary outcome measure. Given the considerable heterogeneity observed in meta-analyses of the impact of EPO administration on transfusion rates in surgical settings, we conducted sensitivity analyses and various subgroup analyses. The sensitivity analysis results demonstrate the robustness of the findings, with the pooled effect size (RR) remaining consistent at 0.51–0.78 even after excluding individual studies. It is reported in the literature that the effect of erythropoietin in reducing blood transfusion during cardiac surgery is weaker than that in orthopedic surgery (6). Consistent with the conclusion of our research, in the subgroup analysis of blood transfusion rates for surgical types, the RR for cardiac surgery was 0.68 and that for orthopedic surgery was 0.57. Notably, our comprehensive pooling of RCT data revealed significant variations in EPO interventions concerning dosage, timing of administration, mode of administration, and molecular subtype, encompassing α, β, and darbepoetin. Further, RCTs comprising 320 patients undergoing coronary artery bypass grafting showed that the ultra-short-term (average 2.4 days) high-dose EPO regimen increased postoperative Hb by 15.5% and reduced the need for blood transfusion by 57% (hazard ratio 0.43, *p* = 0.008) (31).

However, these different studies lack standardization in the dosage, administration timing (preoperative/postoperative), and treatment course of EPO, making it difficult to directly compare their results.

These distinct molecular forms of EPO have direct pharmacodynamics effects, potentially influencing transfusion rates among surgical patients.

Our meta-analysis included subgroup analyses of primary outcomes, presenting pooled EPO molecular types and surgical types, as well as subgroup analyses of transfusion rates based on EPO molecular type. We expanded the analysis of surgical types to include a novel gynecologic abdominal surgery. However, the low RR value for gynecological surgery, attributed to a small sample size, renders the results unreliable. Future analyses will incorporate additional samples from forthcoming clinical reports.

Epoetin alfa and epoetin beta—first-generation short-acting EPOs—act by binding directly to the EPO receptor, stimulating erythrocyte precursor cell proliferation and differentiation. Epoetin alfa and epoetin beta function by mimicking the action of endogenous EPO, a hormone primarily produced by the kidneys in response to hypoxia. These recombinant proteins bind to the EPO receptor on erythroid progenitor cells, activating intracellular signaling pathways such as JAK2/STAT5, RAS/MEK/ERK, and PI3K. This activation leads to the proliferation and differentiation of erythroid precursors into mature red blood cells, thereby alleviating anemia (32, 33). Darbepoetin alfa, a second-generation long-acting EPO analog, exhibits prolonged receptor interaction owing to its high sialic acid content and slow metabolic clearance. Notably, this study does not encompass third-generation EPOs such as methoxy PEG-EPO beta (C.E.R.A.). Our findings indicate that both beta and alpha significantly decreased transfusion rates (RR: 0.58–0.65), whereas darbepoetine did not demonstrate a significant effect. Nonetheless, the limited inclusion of only 2 RCTs in the darbepoetine subgroup, owing to its small sample size, compromised the reliability of the subgroup analysis. More high-quality RCTs should be included in future analyses. Discrepancies in heterogeneity among subgroups could potentially be attributed to variations in drug half-life, dosing regimen, or study design. We intend to remain vigilant for forthcoming literature in this area, particularly for future investigations incorporating a more comprehensive selection of clinically relevant trials on erythropoietin (EPO) molecular variants for meta-analytical purposes, representing our prospective research focus.

While EPO may offer substantial benefits to surgical patients, the primary focus of this study is to assess its impact on reducing allogeneic transfusion rates in this population. Additionally, the cost-effectiveness of blood transfusions and erythropoietin warrants careful consideration. The price of red blood cell suspension ranges from $240.90 to $251.18 per unit, based on data from 2018 to 2019 (34). According to the updated AABB international guidelines in 2023, the average procurement cost of RBC in the United States is 215 dollars (35). In the erythropoietin group (*n* = 339), the average red blood cell usage was 0.50 units (U) per patient, with a blood transfusion rate of 16%. In contrast, the control group (*n* = 344) exhibited an average usage of 0.71 U per patient and a transfusion rate of 26%. Consequently, erythropoietin resulted in an average reduction of 29% in red blood cell usage (odds, 0.71; 95% CI, 0.42–1.13) and a 50% reduction in transfusion patients (odds ratio, 0.5; 95% CI, 0.35–0.75). Additionally, the use of erythropoietin increased the cost per patient by 785 euros (95% CI, 262–1,309), translating to a total of 7,300 euros (95% CI, 1,900–24,000) for each avoided blood transfusion (36). Erythropoietin can decrease the need for blood transfusions; however, its cost is a significant consideration. Indirect costs are often overlooked: while EPO may reduce the length of hospital stays, the associated indirect benefits, such as the time required to return to work, are frequently excluded from most studies. Epoetin-α has been shown to increase preoperative hemoglobin levels and reduce the need for blood transfusions; it also shortens patients' hospital stays (37). A simulation study involving 50,000 patients, based on controlled trial data, demonstrated that preoperative administration of erythropoietin is more cost-effective than autologous or allogeneic blood transfusion (38). A review of the literature identifies the following key factors influencing the cost-effectiveness of EPO in reducing blood transfusions: (1) patient selection: EPO is particularly effective in patients with preoperative anemia (hemoglobin levels of 10–13 g/dl); (2) transfusion threshold: implementing restrictive transfusion strategies, such as maintaining hemoglobin levels below 7 g/dl, can lower the overall demand for EPO (39); and (3) regional differences: in resource-limited areas, such as India, the utilization of EPO remains low owing to its high cost, leading to a greater reliance on lower hemoglobin thresholds (< 7 g/dl) for blood transfusions.

Nevertheless, a thorough evaluation of EPO usage is warranted owing to potential adverse reactions associated with its administration. Therefore, in addition to subgroup analyses based on the type of surgery and analytical approach, this study integrates various outcome measures such as average blood transfusion rates, mortality, postoperative infection rates, postoperative complications, adverse reactions, thrombosis incidence, and length of hospital stay to enhance the comprehensive assessment of EPO's effects on perioperative patients. We systematically assessed the safety indicators associated with the EPO group, incorporating them as secondary outcomes in our analysis. These indicators included mortality rate, postoperative infection rate, postoperative complications and adverse reactions, incidence of venous thromboembolic events (VTE), and length of hospital stay. The results indicated no significant differences in these safety indicators between the EPO group and the control group. While EPO significantly decreased the blood transfusion rate, data on safety, particularly regarding thrombotic events, were limited, and only a few studies addressed cost-effectiveness.

This study focused on the use of EPO during the perioperative period. Besides the above factors, it is also necessary to consider identifying a specific patient group. The updated management of severe perioperative bleeding in 2023, such as the guidelines of the European Society of Anesthesiology and Critical Care (ESAIC), recommend the use of EPO as follows: (1) if anemia is present before surgery and other causes (such as autoimmunity, bone marrow dysfunction, nutritional deficiencies) have been ruled out or treated, it is recommended to use erythropoietin stimulants; (2) we recommend the combined use of erythropoietin and iron supplements for patients with gynecological conditions who have iron deficiency anemia. For all types of patients with gynecological cancers, we recommend the use of tranexamic acid to reduce perioperative bleeding; (3) for anemic patients with postoperative hemoglobin levels lower than 10 g/dl, it is recommended that after excluding contraindications, timely intravenous iron therapy be administered based on body weight. We suggest considering the use of erythropoietin stimulants as adjuvant therapy; (4) for cases where erythropoietin stimulation is administered intravenously and the cause of anemia is simple, we recommend a treatment interval of 1–2 weeks; (5) for patients undergoing hip or knee replacement surgery, if the preoperative hemoglobin concentration is lower than 12 g/dl (for females) or 13 g/dl (for males), treatment with oral or intravenous iron supplements combined with EPO should be performed based on the blood preservation algorithm; (6) for anemia patients receiving orthopedic surgery who are anemic, in whom nutritional deficiencies have been corrected or do not exist, it is recommended to use erythropoietin (ESAs); and (7) intravenous injection of iron or erythropoietin for the treatment of postpartum anemia. The presence of anemia before delivery is an important risk factor for postpartum anemia (40). Regarding the determination of perioperative blood transfusion recipients, the free blood transfusion protocol with a blood transfusion threshold set at 9–10 g/day is the most common, while the restrictive blood transfusion protocol with a threshold of 7–8 g/day is more common. As a reference for red blood cell transfusion, the 2023 AABB international guidelines have the following recommendations: recommendation 1: according to the restrictive blood transfusion threshold standards adopted in most clinical trials, clinicians can set a threshold of 7.5 g/min for patients undergoing cardiac surgery, while for patients undergoing orthopedic surgery or those with pre-existing cardiovascular diseases, a threshold of 8 g/min should be set. Recommendation 2: for children with hemodynamically stable congenital heart disease, the international expert group suggests setting blood transfusion thresholds based on the type of cardiac malformation and the stage of surgical repair: 7 g/dl for biventricular repair and 9 g/dl for single-ventricular palliative care (35). Clarifying the beneficiary patient group is an important direction for long-term research. “Identifying the optimal beneficiary population” is an important research direction for us in the future.

Our review has several limitations. First, the extensive inclusion of literature spanning from 1994 to 2024, a 30-year period, may introduce variability because of changes in surgical practices and blood management strategies over time, leading to differing evaluations of the intervention's effectiveness. Second, the heterogeneity of our results is constrained by variations in erythropoietin dosage, timing, administration methods, and outcome reporting across trials. Despite employing random effects models in meta-analyses to address this issue, subgroup analyses were conducted but continued to exhibit persistent heterogeneity. Heterogeneity arises from variations in surgical types, treatment regimens, the quality of included RCTs, and limitations in sample sizes; some studies, for instance, involve relatively small cohorts, such as a retrospective analysis of only 29 patients. The limitations of outcome measures can be attributed to several factors: (1) the blood transfusion threshold is inconsistent, as different studies employ varying definitions of transfusion indications based on hemoglobin levels, clinical symptoms, or ASA scores, resulting in divergent evaluations of the effects of erythropoietin administration; (2) the clinical criteria for intraoperative or postoperative blood transfusion are established based on perioperative and postoperative hemoglobin levels, ASA scores, and/or clinical symptoms indicative of anemia; and (3) there is a lack of comprehensive secondary outcome data; while EPO has been shown to significantly reduce the blood transfusion rate, reports of adverse reactions are scarce, and only a limited number of studies have assessed cost-effectiveness.

Variations in bleeding patterns among patients undergoing different surgeries necessitate caution in providing standardized recommendations. Further research is essential to establish uniform guidelines for EPO dosage, timing, and administration methods to enhance the comprehensive assessment of its effects on transfusion rates in surgical settings. While the studies analyzed in this review are predominantly RCTs, they exhibit limitations in blinding, allocation concealment, and outcome assessment. Although potential selection bias cannot be entirely discounted, efforts were made to address this through Rabe and funnel plots. Funnel plot asymmetry indicates the possible existence of unpublished small-sample negative results, yet no significant bias was detected. Future investigations will aim to incorporate higher-quality literature to impartially assess the impact of EPO on surgical patients.

Existing evidence indicates that EPO is effective in reducing the need for allogeneic blood transfusions—however, methodological limitations, including risk of bias, heterogeneity, and a lack of cost data—restrict the generalizability of this conclusion. Recent literature suggests that advancements in synthesizing EPO receptors through gene editing and small-molecule induction could significantly lower the cost of cytokines in cultured red blood cells, potentially challenging the economic viability of traditional EPO in the future (41).

The efficacy of erythropoietin administration in reducing allogeneic blood transfusion relies on high-quality RCTs and standardized reporting practices. Future research should focus on increasing sample sizes, standardizing endpoint indicators, and investigating cost optimization strategies, such as reducing the duration of EPO treatment or integrating new synthetic receptor technologies. In clinical practice, it is advisable to consider the patient's baseline hemoglobin levels, the type of surgery, and multidisciplinary blood management protocols to attain the optimal benefit-risk ratio.

By conducting a comprehensive analysis that considers patient prognosis, medical institutions and clinicians can make informed decisions regarding the use of EPO to minimize blood transfusion requirements in surgical patients, leading to more holistic and cost-effective conclusions.

## 5 Conclusion

This study conducted a comprehensive assessment of the efficacy (transfusion rate, average transfusion volume) and safety (side effects, adverse reactions, infection rate, mortality, thrombotic events, length of hospital stay) of erythropoietin intervention during the perioperative period in surgical patients through meta-analysis. The findings indicate that EPO intervention significantly decreases both transfusion rate and average transfusion volume. Subgroup analyses based on the type of surgery revealed consistent outcomes for cardiac and orthopedic procedures, demonstrating reduced transfusion requirements. However, individualized decision-making is necessary for gastrointestinal surgery, considering factors such as the patient's bleeding risk. Additionally, further high-quality studies of gynecological surgery are warranted to corroborate existing results. Subgroup analysis by EPO molecule type demonstrated that both EPO-βand EPO-α effectively reduced transfusion requirements, while adjustments in dosage or target population may be necessary for darbepoetin.

No significant differences were noted in postoperative mortality, complications, side effects, adverse events, venous thromboembolic events (including deep vein thrombosis and pulmonary embolism), and length of hospital stay as secondary outcome measures. Surprisingly, erythropoietin administration exhibited a dual effect by reducing transfusion and infection rates among patients receiving orthopedic surgery without increasing infection risk in other patient groups, suggesting its safe use for transfusion reduction. While the study did not reveal significant variances in postoperative outcomes, the potential impact of EPO intervention on surgical patients' prognosis warrants attention. Further clinical trials are imperative to validate these findings.

## References

[1] Weiser TG Haynes AB Molina G Lipsitz SR Esquivel MM Uribe-Leitz T . Size and distribution of the global volume of surgery in 2012. Bull World Health Organ. (2016) 94:201–9F. doi: 10.2471/BLT.15.159293 26966331 PMC4773932

[2] He YK Li HZ. Lu HD. Is blood transfusion associated with an increased risk of infection among spine surgery patients?: a meta-analysis. Medicine. (2019) 98:e16287. doi: 10.1097/MD.0000000000016287 31305412 PMC6641843

[3] Halmin M Chiesa F Vasan SK Wikman A Norda R Rostgaard K . Epidemiology of massive transfusion: a binational study from Sweden and Denmark. Crit Care Med. (2016) 44:468–77. doi: 10.1097/CCM.0000000000001410 26901542

[4] Coccolini F Shander A Ceresoli M Moore E Tian B Parini D . Strategies to prevent blood loss and reduce transfusion in emergency general surgery, WSES-AAST consensus paper. World J Emerg Surg. (2024) 19:26. doi: 10.1186/s13017-024-00554-7 39010099 PMC11251377

[5] Alsaleh K Alotaibi GS Almodaimegh HS Aleem AA Kouroukis CT. The use of preoperative erythropoiesis-stimulating agents (ESAS) in patients who underwent knee or hip arthroplasty: a meta-analysis of randomized clinical trials. J Arthroplasty. (2013) 28:1463–72. doi: 10.1016/j.arth.2013.01.024 23528548

[6] Cho BC Serini J Zorrilla-Vaca A Scott MJ Gehrie EA Frank SM . Impact of preoperative erythropoietin on allogeneic blood transfusions in surgical patients: results from a systematic review and meta-analysis. Anesth Analg. (2019) 128:981–92. doi: 10.1213/ANE.0000000000004005 30649068

[7] Aydin Z Mallat MJ Schaapherder AF van Zonneveld AJ van Kooten C Rabelink TJ . Randomized trial of short-course high-dose erythropoietin in donation after cardiac death kidney transplant recipients. Am J Transplant. (2012) 12:1793–800. doi: 10.1111/j.1600-6143.2012.04019.x 22429395

[8] Cao SL Ren Y Li Z Lin J Weng XS Feng B. Clinical effectiveness of 3 days preoperative treatment with recombinant human erythropoietin in total knee arthroplasty surgery: a clinical trial. QJM. (2020) 113:245–52. doi: 10.1093/qjmed/hcz261 31605493

[9] Kim JE Song SW Kim JY Lee HJ Chung KH Shim YH. Effect of a single bolus of erythropoietin on renoprotection in patients undergoing thoracic aortic surgery with moderate hypothermic circulatory arrest. Ann Thorac Surg. (2016) 101:690–6. doi: 10.1016/j.athoracsur.2015.08.007 26576750

[10] Dardashti A Ederoth P Algotsson L Brondén B Grins E Larsson M . Erythropoietin and protection of renal function in cardiac surgery (the EPRICS trial). Surv Anesthesiol. (2014) 59:163–4. doi: 10.1097/01.SA.0000466255.62092.ff 25225746

[11] Urena M Del Trigo M Altisent OA Campelo-Prada F Regueiro A DeLarochelliere R . Combined erythropoietin and iron therapy for anaemic patients undergoing transcatheter aortic valve implantation: the epicure randomised clinical trial. EuroIntervention. (2017) 13:44–52. doi: 10.4244/EIJ-D-16-00591 28067195

[12] Gaston KE Kouba E Moore DT Pruthi RS. The use of erythropoietin in patients undergoing radical prostatectomy: effects on hematocrit, transfusion rates and quality of life. Urol Int. (2006) 77:211–5. doi: 10.1159/000094811 17033207

[13] Scott SN Boeve TJ McCulloch TM Fitzpatrick KA Karnell LH. The effects of epoetin alfa on transfusion requirements in head and neck cancer patients: a prospective, randomized, placebo-controlled study. Laryngoscope. (2002) 112:1221–9. doi: 10.1097/00005537-200207000-00015 12169903

[14] Wurnig C Schatz K Noske H Hemon Y Dahlberg G Josefsson G . Subcutaneous low-dose epoetin beta for the avoidance of transfusion in patients scheduled for elective surgery not eligible for autologous blood donation. Eur Surg Res. (2001) 33:303–10. doi: 10.1159/000049723 11805389

[15] Sowade O Ziemer S Sowade B Franke W Messinger D Ziebell E . The effect of preoperative recombinant human erythropoietin therapy on platelets and hemostasis in patients undergoing cardiac surgery. J Lab Clin Med. (1997) 129:376–83. doi: 10.1016/S0022-2143(97)90186-4 9042823

[16] Sowade O Warnke H Scigalla P Sowade B Franke W Messinger D . Avoidance of allogeneic blood transfusions by treatment with epoetin beta (recombinant human erythropoietin) in patients undergoing open-heart surgery. Blood. (1997) 89:411–8. doi: 10.1182/blood.V89.2.411 9002942

[17] Hayashi J Kumon K Takanashi S Kawashima Y Eguchi S Takaku F . Subcutaneous administration of recombinant human erythropoietin before cardiac surgery: a double-blind, multicenter trial in japan. Transfusion. (1994) 34:142–6. doi: 10.1046/j.1537-2995.1994.34294143943.x 8310485

[18] Faris PM Ritter MA Abels RI. The effects of recombinant human erythropoietin on perioperative transfusion requirements in patients having a major orthopaedic operation. The American Erythropoietin Study Group. J Bone Joint Surg Am. (1996) 78:62–72. doi: 10.2106/00004623-199601000-00009 8550681

[19] Feagan BG Wong CJ Kirkley A Johnston DW Smith FC Whitsitt P . Erythropoietin with iron supplementation to prevent allogeneic blood transfusion in total hip joint arthroplasty. A randomized, controlled trial. Ann Intern Med. (2000) 133:845–54. doi: 10.7326/0003-4819-133-11-200012050-00008 11103054

[20] Weber EW Slappendel R Hemon Y Mahler S Dalen T Rouwet E . Effects of epoetin alfa on blood transfusions and postoperative recovery in orthopaedic surgery: the European Epoetin Alfa Surgery Trial (EEST). Eur J Anaesthesiol. (2005) 22:249–57. doi: 10.1017/S0265021505000426 15892401

[21] Kettelhack C Hones C Messinger D Schlag PM. Randomized multicentre trial of the influence of recombinant human erythropoietin on intraoperative and postoperative transfusion need in anaemic patients undergoing right hemicolectomy for carcinoma. Br J Surg. (1998) 85:63–7. doi: 10.1046/j.1365-2168.1998.00564.x 9462386

[22] Norager CB Jensen MB Madsen MR Qvist N Laurberg S. Effect of darbepoetin alfa on physical function in patients undergoing surgery for colorectal cancera randomized, double-blind, placebo-controlled study. Oncology. (2006) 71:212–20. doi: 10.1159/000106071 17641543

[23] Na HS Shin SY Hwang JY Jeon YT Kim CS Do SH. Effects of intravenous iron combined with low-dose recombinant human erythropoietin on transfusion requirements in iron-deficient patients undergoing bilateral total knee replacement arthroplasty (CME). Transfusion. (2011) 51:118–24. doi: 10.1111/j.1537-2995.2010.02783.x 20624257

[24] Christodoulakis M Tsiftsis DD Hellenic Surgical Oncology Perioperative EPOSG. Preoperative epoetin alfa in colorectal surgery: a randomized, controlled study. Ann Surg Oncol. (2005) 12:718–25. doi: 10.1245/ASO.2005.06.031 16052276

[25] D'Ambra MN Gray RJ Hillman R Jones JW Kim HC Rawitscher R . Effect of recombinant human erythropoietin on transfusion risk in coronary bypass patients. Ann Thorac Surg. (1997) 64:1686–93. doi: 10.1016/S0003-4975(97)00839-4 9436556

[26] Kim JH Shim JK Song JW Song Y Kim HB Kwak YL. Effect of erythropoietin on the incidence of acute kidney injury following complex valvular heart surgery: a double blind, randomized clinical trial of efficacy and safety. Crit Care. (2013) 17:R254. doi: 10.1186/cc13081 24156702 PMC4056185

[27] Dousias V Paraskevaidis E Dalkalitsis N Tsanadis G Navrozoglou I Lolis D. Recombinant human erythropoietin in mildly anemic women before total hysterectomy. Clin Exp Obstet Gynecol. (2003) 30:235–8. 14664421

[28] Kong R Hutchinson N Hill A Ingoldby F Skipper N Jones C . Randomised open-label trial comparing intravenous iron and an erythropoiesis-stimulating agent versus oral iron to treat preoperative anaemia in cardiac surgery (initiate trial). Br J Anaesth. (2022) 128:796–805. doi: 10.1016/j.bja.2022.01.034 35256150

[29] Laupacis A Feagan B Wong C. Effectiveness of perioperative recombinant human erythropoietin in elective hip replacement. COPES Study Group. Lancet. (1993) 342:378. doi: 10.1016/0140-6736(93)91527-S 8101624

[30] Kosmadakis N Messaris E Maris A Katsaragakis S Leandros E Konstadoulakis MM . Perioperative erythropoietin administration in patients with gastrointestinal tract cancer: prospective randomized double-blind study. Ann Surg. (2003) 237:417–21. doi: 10.1097/01.SLA.0000055275.38740.56 12616127 PMC1514310

[31] Weltert L D'Alessandro S Nardella S Girola F Bellisario A Maselli D . Preoperative very short-term, high-dose erythropoietin administration diminishes blood transfusion rate in off-pump coronary artery bypass: a randomized blind controlled study. J Thorac Cardiovasc Surg. (2010) 139:621–6. discussion: 626–7. doi: 10.1016/j.jtcvs.2009.10.012 20042202

[32] Kuhrt D Wojchowski DM. Emerging EPO and EPO receptor regulators and signal transducers. Blood. (2015) 125:3536–41. doi: 10.1182/blood-2014-11-575357 25887776 PMC4458796

[33] Rainville N Jachimowicz E Wojchowski DM. Targeting EPO and EPO receptor pathways in anemia and dysregulated erythropoiesis. Expert Opin Ther Targets. (2016) 20:287–301. doi: 10.1517/14728222.2016.1090975 26419263 PMC4829957

[34] Indelen C Uygun Kizmaz Y Kar A Shander A Kirali K. The cost of one unit blood transfusion components and cost-effectiveness analysis results of transfusion improvement program. Turk Gogus Kalp Damar Cerrahisi Derg. (2021) 29:150–7. doi: 10.5606/tgkdc.dergisi.2021.20886 34104508 PMC8167483

[35] Carson JL Stanworth SJ Guyatt G Valentine S Dennis J Bakhtary S . Red blood cell transfusion: 2023 AABB international guidelines. JAMA. (2023) 330:1892–902. doi: 10.1001/jama.2023.12914 37824153

[36] So-Osman C Nelissen RG Koopman-van Gemert AW Kluyver E Pöll RG Onstenk R . Patient blood management in elective total hip- and knee-replacement surgery (Part 1): a randomized controlled trial on erythropoietin and blood salvage as transfusion alternatives using a restrictive transfusion policy in erythropoietin-eligible patients. Anesthesiology. (2014) 120:839–51. doi: 10.1097/ALN.0000000000000134 24424070

[37] Delasotta LA Rangavajjula A Frank ML Blair J Orozco F Ong A. The use of preoperative epoetin-α in revision hip arthroplasty. Open Orthop J. (2012) 6:179–83. doi: 10.2174/1874325001206010179 22629289 PMC3358717

[38] Tomeczkowski J Stern S Müller A von Heymann C. Potential cost saving of epoetin alfa in elective hip or knee surgery due to reduction in blood transfusions and their side effects: a discrete-event simulation model. PLoS ONE. (2013) 8:e72949. doi: 10.1371/journal.pone.0072949 24039829 PMC3767728

[39] Spahn DR Schoenrath F Spahn GH Seifert B Stein P Theusinger OM . Effect of ultra-short-term treatment of patients with iron deficiency or anaemia undergoing cardiac surgery: a prospective randomised trial. Lancet. (2019) 393:2201–12. doi: 10.1016/S0140-6736(18)32555-8 31036337

[40] Kietaibl S Ahmed A Afshari A Albaladejo P Aldecoa C Barauskas G . Management of severe peri-operative bleeding: guidelines from the European Society of Anaesthesiology and Intensive Care: second update 2022. Eur J Anaesthesiol. (2023) 40:226–304. doi: 10.1097/EJA.0000000000001803 36855941

[41] Shah AP Majeti KR Ekman FK Selvaraj S Sharma D Sinha R . Engineering synthetic signaling receptors to enable erythropoietin-free erythropoiesis. Nat Commun. (2025) 16:1140. doi: 10.1038/s41467-025-56239-5 39880867 PMC11779867

[42] Dousias V Stefos T Navrozoglou I Staikos I Ditto A Paraskevaidis E. Administration of recombinant human erythropoietin in patients with gynecological cancer before radical surgery. Clin Exp Obstet Gynecol. (2005) 32:129–31. 16108399

[43] Haljan G Maitland A Buchan A Arora RC King M Haigh J . The erythropoietin neuroprotective effect: assessment in CABG surgery (TENPEAKS): a randomized, double-blind, placebo controlled, proof-of-concept clinical trial. Stroke. (2009) 40:2769–75. doi: 10.1161/STROKEAHA.109.549436 19556536

[44] Heiss MM Tarabichi A Delanoff C Allgayer H Jauch KW Hernandez-Richter T . Perisurgical erythropoietin application in anemic patients with colorectal cancer: a double-blind randomized study. Surgery. (1996) 119:523–7. doi: 10.1016/S0039-6060(96)80261-3 8619207

[45] Kateros K Sakellariou VI Sofianos IP Papagelopoulos PJ. Epoetin alfa reduces blood transfusion requirements in patients with intertrochanteric fracture. J Crit Care. (2010) 25:348–53. doi: 10.1016/j.jcrc.2009.04.008 19592206

[46] Luchette FA Pasquale MD Fabian TC Langholff WK Wolfson M. A randomized, double-blind, placebo-controlled study to assess the effect of recombinant human erythropoietin on functional outcomes in anemic, critically ill, trauma subjects: the long term trauma outcomes study. Am J Surg. (2012) 203:508–16. doi: 10.1016/j.amjsurg.2011.08.006 22177550

[47] Qvist N Boesby S Wolff B Hansen CP. Recombinant human erythropoietin and hemoglobin concentration at operation and during the postoperative period: reduced need for blood transfusions in patients undergoing colorectal surgery—prospective double-blind placebo-controlled study. World J Surg. (1999) 23:30–5. doi: 10.1007/s002689900561 9841760

[48] Weltert L Rondinelli B Bello R Falco M Bellisario A Maselli D . A single dose of erythropoietin reduces perioperative transfusions in cardiac surgery: results of a prospective single-blind randomized controlled trial. Transfusion. (2015) 55:1644–54. doi: 10.1111/trf.13027 25702777

[49] Yoo YC Shim JK Kim JC Jo YY Lee JH Kwak YL. Effect of single recombinant human erythropoietin injection on transfusion requirements in preoperatively anemic patients undergoing valvular heart surgery. Surv Anesthesiol. (2012) 56:106–7. doi: 10.1097/01.SA.0000413395.98867.60 22027622

[50] Totonchi Z Noohi F Futuhi F Azarfarin R Radbin P. Effects of recombinant erythropoietin on hemoglobin levels and blood transfusion needs in patients with preoperative anemia undergoing cardiac surgery. Ann Card Anaesth. (2022) 25:466–71. doi: 10.4103/aca.aca_42_21 36254912 PMC9732958

[51] Saour M Blin C Zeroual N Mourad M Amico M Gaudard P . Impact of a bundle of care (intravenous iron, erythropoietin and transfusion metabolic adjustment) on post-operative transfusion incidence in cardiac surgery: a single-centre, randomised, open-label, parallel-group controlled pilot trial. Lancet Reg Health Eur. (2024) 43:100966. doi: 10.1016/j.lanepe.2024.100966 39022429 PMC11254177

[52] Yazicioglu L Eryilmaz S Sirlak M Inan MB Aral A Tasoz R . Recombinant human erythropoietin administration in cardiac surgery. J Thorac Cardiovasc Surg. (2001) 122:741–5. doi: 10.1067/mtc.2001.115426 11581607

[53] Van Loo A Vanholder R Bernaert P De Roose J Lameire N. Recombinant human erythropoietin corrects anaemia during the first weeks after renal transplantation: a randomized prospective study. Nephrol Dial Transplant. (1996) 11:1815–21. doi: 10.1093/oxfordjournals.ndt.a027674 8918628

