# Supplementary material for: Effects of erythropoietin administration on allogeneic blood transfusion requirements in adults undergoing surgery: a systematic review and meta-analysis
Source: Front Med (Lausanne). 2026 Jan 14;12:1712121. doi: 10.3389/fmed.2025.1712121 (PMC12847391; doi:10.3389/fmed.2025.1712121)
Supplement: Supplementary file 1 [file Table_1.doc]

**Electronic Supplementary Material**

The search strategy of the PubMed database ： ((((((((adult) OR (Aged)) OR (adult *)) OR (aged)) OR (over 18)) OR (>18)) AND ((((( ( ((((((((((((((Erythropoietin) OR (Erythropoietin, Recombinant)) OR (erythropoietin[Title/Abstract])) OR (EPO)) OR (epo)) OR (rhEPO)) OR (Recombinant Human Erythropoietin)) OR (erythropoiesis-stimulating agents)) OR (Epogen)) OR (Eprex)) OR (eporatio)) OR (Epoietin)) OR (exp Erythropoietin/)) OR (Espogen)) OR (Espogen)) OR (Epofit)) OR (Erykine)) OR (EPOTrust)) OR (exp erythropoietin/)) OR (Erythropoi*))) AND ((((((Surgical Procedures, Operative ) OR (Operative Procedures)) OR (Operative Procedure)) OR ((((((((((((((((Surgical Procedures, Operative ) OR (Operative Procedures)) OR (Operative Procedure)) OR (Procedure, Operative)) OR (Procedures, Operative)) OR (Operative Surgical Procedure)) OR (Operative Surgical Procedures)) OR (Procedure, Operative Surgical)) OR (Procedures, Operative Surgical)) OR (Surgical Procedure, Operative)) OR (Surgical Procedures)) OR (Procedures, Surgical)) OR (.Procedure, Surgical)) OR (.Surgical Procedure)) OR (Surgery, Ghost)) OR (Ghost Surgery))) OR ((((((General Surgery) OR (Surgery, General)) OR (Surgery)) OR (Surg*)) OR (Operat*)) OR (Perioperat*))) OR ((((((Preoperative Care) OR (Care, Preoperative)) OR (Preoperative Procedure)) OR (Preoperative Procedures)) OR (Procedure, Preoperative)) OR (Procedures, Preoperative)))) AND (((((((Blood Transfusion) OR (Blood Transfusions)) OR (Transfusion, Blood)) OR (Transfusions, Blood)) AND ((((Blood Transfusion) OR (Blood Transfusions)) OR (Transfusion, Blood)) OR (Transfusions, Blood))) OR (((((((((Erythrocyte Transfusion) OR (Erythrocyte Transfusions) ) OR (Transfusion, Erythrocyte)) OR (Transfusions, Erythrocyte)) OR (Red Blood Cell Transfusions)) OR (Transfusion, Red Blood Cell)) OR (Transfusions, Red Blood Cell)) OR (Red Blood Cell Transfusio)) OR (RBC transfusion))) OR (allogeneic blood transfusion)) Filters: Randomized Controlled Trial, subsequently refining it based on the glossaries of each database (for details, see S1 File ).

**Pubmed , Web of Science , EMBASE, Cochrane databases Search Strategy**

1. adult

2. Aged

3. adult *

4. aged

5. over 18

6. >18

7. 1 or 2 or 3 or 4 or 5 or 6

8.Erythropoietin

9.Erythropoietin, Recombinant

10.erythropoietin

11.EPO

12.epo

13.rhEPO

14.Recombinant Human Erythropoietin

15.erythropoiesis-stimulating agents

16.Epogen

17.Eprex

18.eporatio

19.Epoietin

20.exp Erythropoietin/

21.Espogen

22.Erypro

23.Epofit

24.Erykine

25.EPOTrust

26.exp erythropoietin/

27.Erythropoi*

28.8 or 9 or 10 or 11 or 12 or 13 or 14 or 15 or 16 or 17 or 18 or 19 or 20 or 21 or 22 or 23 or 24 or 25 or 26 or 27

29.Surgical Procedures, Operative

30.Operative Procedures

31.Operative Procedure

32.Procedure, Operative

33.Procedures, Operative

34.Operative Surgical Procedure

35.Operative Surgical Procedures

36.Procedure, Operative Surgical

37.Procedures, Operative Surgical

38.Surgical Procedure, Operative

39.Surgical Procedures

40.Procedures, Surgical

41.Procedure, Surgical

42.Surgical Procedure

43.Surgery, Ghost

44.Ghost Surgery

45.29 or 30 or 31 or 32 or 33 or 34 or 35 or 36 or 37 or 38 or 39 or 40 or 41 or 42 or 43 or 44

46.General Surgery

47.Surgery, General

48.Surgery

49.Surg*

50.Operat*

51.Perioperat*

52.46 or 47 or 48 or 49 50 or 51

53.Preoperative Care

54.Care, Preoperative

55.Preoperative Procedure

56.Preoperative Procedures

57.Procedure, Preoperative

58.Procedures, Preoperative

59.53 or 54 or 55 or 56 or 57 or 58

60.45 or 52 or 59

61.Blood Transfusion

62.Blood Transfusions

63.Transfusion, Blood

64.Transfusions, Blood

65.60 or 61 or 62 or 63

66.Erythrocyte Transfusion

67.Erythrocyte Transfusions

68.Transfusion, Erythrocyte

69.Transfusions, Erythrocyte

70.Red Blood Cell Transfusions

71.Transfusion, Red Blood Cell

72.Transfusions, Red Blood Cell

73.Red Blood Cell Transfusio

74.RBC transfusion

75. 66 or 67 or 68 or 69 or 70 or 71 or 72 or 73 or74

76.allogeneic blood transfusion

77.65 or 75 or 76

78.randomized controlled trial.pt. or randomized controlled trial.mp.

79.7 and 28 and 60 and 77 and 78
