# Supplementary material for: Effects of erythropoietin administration on allogeneic blood transfusion requirements in adults undergoing surgery: a systematic review and meta-analysis
Source: Front Med (Lausanne). 2026 Jan 14;12:1712121. doi: 10.3389/fmed.2025.1712121 (PMC12847391; doi:10.3389/fmed.2025.1712121)
Supplement: Supplementary file 2 [file Table_2.docx]

**S2 Table. GRADE Assessment****.**

| Outcomes | Study  Design | Risk of Bias | Inconsistency | Indirectness | Imprecision | Other  Considerations | Quality |
| --- | --- | --- | --- | --- | --- | --- | --- |
|  | | | | | | |  |
| The perioperative allogeneic red blood cell transfusion rate | RCTs | Serious | Serious | No | No | No | Low |
| The number of allogeneic red blood cell transfusions | RCTs | Serious | Serious | No | No | No | Low |
| Mortality | RCTs | Serious | No | No | Serious | No | Low |
| Postoperative infection rate | RCTs | Serious | No | No | Serious | No | Low |
| Postoperative complications and adverse effects | RCTs | Serious | No | No | Serious | No | Low |
| Venous thromboembolic events (VTE) rate | RCTs | Serious | No | No | Serious | No | Low |
| Hospital length of stay | RCTs | Serious | Serious | No | Serious | No | Very low |
